# Comprehensive analysis of MAPK gene family in upland cotton (*Gossypium hirsutum*) and functional characterization of *GhMPK31* in regulating defense response to insect infestation

**DOI:** 10.1007/s00299-024-03167-1

**Published:** 2024-03-18

**Authors:** Fuqiu Wang, Sijia Liang, Guanying Wang, Qiongqiong Wang, Zhongping Xu, Bo Li, Chunyang Fu, Yibo Fan, Tianyu Hu, Muna Alariqi, Amjad Hussain, Jinglin Cao, Jian Li, Xianlong Zhang, Shuangxia Jin

**Affiliations:** 1 Hubei Hongshan Laboratory, National Key Laboratory of Crop Genetic Improvement, Huazhong Agricultural University, Wuhan, 430070 China; 2 Academy of Industry Innovation and Development, Huanghuai University, Zhumadian, 463000 Henan China; 3 Tobacco Research Institute of Hubei Province, Wuhan, 430030 Hubei People’s Republic of China; 4 The Southern Xinjiang Research Institute of Shihezi University, TuMu ShuKe, Xinjiang, 843900 China

**Keywords:** Cotton, *MAPK* gene family, HR-like cell death, H_2_O_2_, Insect resistance

## Abstract

**Key message:**

The transcriptomic, phenotypic and metabolomic analysis of transgenic plants overexpressing *GhMPK31* in upland cotton revealed the regulation of H_2_O_2_ burst and the synthesis of defensive metabolites by *GhMPK31*.

**Abstract:**

Mitogen-activated protein kinases (MAPKs) are a crucial class of protein kinases, which play an essential role in various biological processes in plants. Upland cotton (*G. hirsutum*) is the most widely cultivated cotton species with high economic value. To gain a better understanding of the role of the *MAPK* gene family, we conducted a comprehensive analysis of the *MAPK* gene family in cotton. In this study, a total of 55 *GhMPK* genes were identified from the whole genome of *G. hirsutum*. Through an investigation of the expression patterns under diverse stress conditions, we discovered that the majority of *GhMPK* family members demonstrated robust responses to abiotic stress, pathogen stress and pest stress. Furthermore, the overexpression of *GhMPK31* in cotton leaves led to a hypersensitive response (HR)-like cell death phenotype and impaired the defense capability of cotton against herbivorous insects. Transcriptome and metabolomics data analysis showed that overexpression of *GhMPK31* enhanced the expression of H_2_O_2_-related genes and reduced the accumulation of defensive related metabolites. The direct evidence of GhMPK31 interacting with GhRBOHB (H_2_O_2_-generating protein) were found by Y2H, BiFC, and LCI. Therefore, we propose that the increase of H_2_O_2_ content caused by overexpression of *GhMPK31* resulted in HR-like cell death in cotton leaves while reducing the accumulation of defensive metabolites, ultimately leading to a decrease in the defense ability of cotton against herbivorous insects. This study provides valuable insights into the function of *MAPK* genes in plant resistance to herbivorous insects.

**Supplementary Information:**

The online version contains supplementary material available at 10.1007/s00299-024-03167-1.

## Introduction

MAPK is an important serine/threonine protein kinase which forms a cascade of MAPK by sequential phosphorylation with MAP kinase kinase (MAPKK/MEK) and MAP kinase kinase kinase (MAPKKK/MEKK) (Hamel et al. 2006; Jagodzik et al. 2018). MAPK cascades are found extensively across eukaryotes, exhibiting high levels of conservation throughout evolution, and serving a crucial role in signal transduction pathways in plants (Group 2002). When plants are stimulated by the external environment, MAPKKK undergoes initial phosphorylation by upstream signals, and the activated MAPKKK proceeds to phosphorylates downstream MAPKK by targeting serine/threonine residues within the conserved S/T-X_3-5_-S/T motifs (Pitzschke 2015). Then, Activated MAPKKs phosphorylate downstream MAPKs through tyrosine and threonine residues in the conserved activation loop (T-loop). Finally, activated MAPKs can complete the signal transduction process by phosphorylating various downstream substrates (Zhang et al. 2018). As the terminal element of MAPK cascade, MAPK is responsible for relaying the signal to downstream components. In the face of various substrates such as cytoplasmic protein kinases, cytoplasmic cytoskeleton and nuclear transcription factors (TFs), MAPK shows more complexity and functional diversity (Xu and Zhang 2015).

Previous studies have demonstrated MAPKs involvement in diverse biological processes (Zhang and Zhang 2022). In terms of plant growth and development, *AtMPK3/6* is known to be involved in stomatal development, floral organ abscission, meristem maintenance, early anther development and embryogenesis in *Arabidopsis thaliana* (Cho et al. 2008; Hord et al. 2008; Shao et al. 2020; Wang et al. 2007; Zhang et al. 2017). In the response of plants to abiotic stress, SlMPK1 negatively regulates the heat tolerance of tomatoes through directly phosphorylation of the serine-proline-rich protein homolog (SlSPRH1) (Ding et al. 2018). In the response of plants to pathogen stress, *GhMPK20* negatively regulated the resistance of cotton to *Fusarium oxysporum* (Wang et al. 2018). MAPK can also regulate hypersensitive response (HR), a type of programmed cell death (PCD), which is commonly associated with plant disease resistance (Ren et al. 2002). Endogenous activation of *MAPK* represented by *SIPK*, *Ntf4* and *WIPK* in tobacco can trigger HR-like cell death in the absence of pathogens infection (Jin et al. 2003; Ren et al. 2006). The absence of *AtMPK4* results in the dwarfing of *A. thaliana* during the second to third leaf stage, but no necrotic lesions were observed. Silencing of *GmMPK4s* causes stunting in soybean plants, accompanied by spontaneous cell death on leaves and stems (Liu et al. 2011; Petersen et al. 2000). Silenced *GmMPK6* in soybean resulted in leaf growth retardation and spontaneous cell death, while overexpression of *GmMPK6* also resulted in HR-like cell death in tobacco and *A. thaliana* (Liu et al. 2014). Although the precise mechanism underlying plant dwarfing and HR-like cell death remains uncertain, it is strongly associated with the blast of reactive oxygen species (ROS).

However, an increasing amount of biochemical and genetic evidence suggests that MAPK plays a critical role in plant resistance against herbivores. The activation of MAPK is one of the earliest signaling events in plants when they are attacked by herbivores. Upon herbivore attack, the plant MAPK signaling pathway is activated, leading to alterations in plant hormone levels as well as reshaping of the transcriptome and proteome, which ultimately affect the plant's ability to defend against herbivores (Hettenhausen et al. 2015). Applying *Manduca sexta* oral secretions (OS) to leaf wounds in *Nicotiana attenuata* simulates *M. sexta* feeding and significantly induces the activity of MAPK family members *SIPK* and *WIPK*. When *SIPK* or *WIPK* is silenced, the levels of jasmonic acid (JA) induced by *M. sexta* feeding are significantly reduced. Transcriptome analysis reveals that *SIPK* and *WIPK* mediate the accumulation of many defense-related genes induced by *M. sexta* feeding (Wu et al. 2007). JA and ethylene (ET) play positive roles in regulating plant defenses against herbivores, with 1-aminocyclopropane-1-carboxylic acid synthase (ACS) being the rate-limiting ET biosynthesis (Chae et al. 2003). In *A. thaliana*, AtMPK6 increases the production of ET by directly phosphorylating ACS6 and ACS2 (Liu and Zhang 2004). In tomatoes, silencing *MPK1/2* simultaneously results in a decrease in the levels of JA by lowering the expression of JA biosynthetic genes, thereby promoting better growth of *M. sexta* larva on *MPK1/2* silenced plants (Kandoth et al. 2007). In rice, the silenced *OsMPK3* reduced the herbivore-induced trypsin protease inhibitors (TrypPIs) and JA levels, and improved the performance of striped stem borer (SSB) larvae (Wang et al. 2013).

As the various members of *MAPK* exhibit distinct biological functions, there is increasingly necessary to identify MAPK family members in different species. Currently, 20, 17, 21, 16 and 17 *MAPK* gene members have been identified in *A. thaliana*, *Oryza sativa*, *Camellia sinensis*, *Fagopyrum tataricum* and *Lactuca sativa*, respectively (Colcombet and Hirt 2008; Liu et al. 2022; Reyna and Yang 2006; Wang et al. 2022; Yao et al. 2022). Being one of the most significant cash crops globally, cotton yields a substantial quantity of natural fibers and oils. In this study, we present a comprehensive analysis of *MAPK* gene family in cotton and provide functional analysis of *GhMPK31*. Overexpression of *GhMPK31* in cotton results in a dwarfed plant architecture and HR-like cell death in the leaves, this phenotype was found to be associated with a greater accumulation of hydrogen peroxide (H_2_O_2_). To elucidate the regulatory mechanism of *GhMPK31* on ROS burst in cotton, we observed that *GhMPK31* can induce the expression of *Respiratory Burst Oxidase Homolog B* (*GhRBOHB*), also known as NADPH oxidase, which is one of the main enzymes responsible for extracellular ROS generation in plants (Jalmi and Sinha 2015). Therefore, we speculated that *GhMPK31* plays a role in the regulation of H_2_O_2_ synthesis in cotton by influencing the expression of *GhRBOHB*. In addition, selective and non-selective feeding experiments were conducted on wilt type (WT) and OM31 plants using *Helicoverpa armigera* (cotton bollworm) and *Spodoptera litura*, revealing that overexpression of *GhMPK31* enhances leaf feeding by these insects. This effect could be attributed to the regulatory role of *GhMPK31* in the synthesis of defensive compounds.

In conclusion, the comprehensive analysis of *GhMPKs* gene family and the functional characterization of *GhMPK31* enriched the functional study of MAPK in upland cotton and led the foundation for the improvement of upland cotton varieties.

## Materials and methods

### Genome-wide comprehensive analysis of *GhMPKs* gene family

*G. hirsutum* and *G. raimondii* genomic data were obtained from Cottongen (https://www.cottongen.org/), the genomic data of *A. thaliana* and *O. sativa* were obtained from Ensembl Plants (http://plants.ensembl.org/index.html) and Rice Genome Annotation Project (http://rice.uga.edu/downloads_gad.shtml), respectively. *MAPKs* coding sequences of *A. thaliana*, *O. sativa* and *G. raimondii* were used as query sequences to search the genome of *G. hirsutum* (NBI (Zhang et al. 2015) and HAU (Wang et al. 2019)) by BLASTP program, and the local Hidden Markov Model-based searches (HMMER) were constructed according to their *MAPKs* coding sequences to identify *GhMPKs*. Then, the results of BLASTP and HMMER were compared, redundant sequences were removed, the last upload candidate genes to SMART (http://smart.embl.de/) and CDD (https://www.ncbi.nlm.nih.gov/cdd/) confirmed the existence of conservative domains. The amino acid numbers, molecular weight (MW) and isoelectric point (pI) of the GhMPKs were predicted by the ProtParam tool (https://web.expasy.org/protparam/). The subcellular localization, transmembrane domains and signal peptides of GhMPKs were predicted by DeepLoc-2.0 (https://services.healthtech.dtu.dk/services/DeepLoc-2.0/), DeepTMHMM (https://dtu.biolib.com/DeepTMHMM/) and SignalP-6 (https://biolib.com/DTU/SignalP-6/), respectively.

The phylogenetic tree of the four species (*A. thaliana*, *O. sativa*, *G. hirsutum* and *G. raimondii*) was constructed based on their full-length protein sequence with MEGA 7.0 software by the neighbor-joining (NJ) method, and the phylogenetic tree was visualized through EvolView (https://evolgenius.info//evolview-v2). The conserved motifs of GhMPKs were analyzed by the program of MEME and visualized by Tbtools (Chen et al. 2020). The gene structures of *GhMPKs* were analyzed with GSDS software (http://gsds.gao-lab.org/) by comparing coding sequences (CDSs) with the genome sequence corresponding to the gene.

Analysis of phosphorylation sites was completed by using NetPhos-3.1 (https://services.healthtech.dtu.dk/services/NetPhos-3.1/), and to analyze cis-acting in the promoter region of the *GhMPK* genes the PlantCARE database (http://bioinformatics.psb.ugent.be/webtools/plantcare/html/) was used, collinearity analysis between *G. hirsutum* with other three species was determined by MCScanX software, the visualization of all the above analyses was completed by TBtools. The localization images of *GhMPK* genes on chromosomes and their genome-wide gene duplication events were plotted by Circos software.

### Expression of *GhMPK* genes in different stresses

The expression data of *GhMPK* genes in various tissues and abiotic stresses were downloaded from the transcriptome data of TM-1(Zhang et al. 2015), and the transcriptome data of *GhMPK* gene in response to *H. armigera* OS at different time periods were derived from NCBI Sequence Read Archive (accession number: PRJNA522889) (Si et al. 2020). Transcriptome data for stress of *Verticillium dahliae* was obtained from Ping Qiu’s study (Qiu et al. 2023).

### Plant materials and growing conditions

The cultivated variety Jin668 of *G. hirsutum* was used as the stable conversion material. RNA interference (RNAi) and overexpression transgenic lines of *GhMPK31* (IM31-19, IM31-28) and (OM31-11, OM31-24) were screened by expression levels for subsequent studies, and Jin668 was used as control. All materials were cultivated in the field during the regular growing season following the standard tillage practices of Wuhan. During the winter season, plants were grown in a controlled environment in a greenhouse maintained at 26 °C, under 16 h light/8 h dark photoperiod and 60% relative humidity.

### Vector construction and *agrobacterium*-mediated genetic transformation

The full-length CDS and RNAi fragments of *GhMPK31* were separately amplified and inserted into the pK2GW7 and pHellsgate 4 vectors using the Gateway cloning technology (Karimi et al. 2002; Liang et al. 2021; Luo et al. 2017; Tian et al. 2015; Wesley et al. 2001). The recombinant vectors for overexpression and RNAi were transformed into the cotton (cv. Jin668) hypocotyls by *Agrobacterium tumefaciens* (strain GV3101)-mediated transformation (Jin et al. 2006).

### Analysis of RNA-sequencing data

The seeds of T0 generation OM31-11, OM31-24, IM31-19, IM31-28 and WT plants were germinated simultaneously and cultivated in a greenhouse maintained at 28 °C, under 16 h light/8 h dark photoperiod and 60% relative humidity. After 4 weeks, we measured the expression levels of *GhMPK31* in the leaves of all T1 generation plants. We selected the T1 generation OM31-11 and OM31-24 plants with high *GhMPK31* expression levels and the IM31-19 and IM31-28 plants with low *GhMPK31* expression levels for subsequent experiments. The leaves were removed and divided into two portions, one for transcriptome sequencing and the other for Target-like metabolite assays, with 2 bio-replicates per sample. For RNA sequencing (RNA-seq) reads from different samples, the low-quality reads were filtered using Trimmomatic (v.0.39), and the clean reads were then mapped to the TM-1 reference genome (Zhang et al. 2015) by HISAT2 (v.2.2.1) (Kim et al. 2019) with default parameters. The expression level (transcripts per million; TPM) of genes was calculated by StringTie (v.2.1.4). A gene was considered to be expressed if its TPM was > 0. Subsequently, differentially expressed genes (DEGs) were identified by using the DESeq2 package with False Discovery Rate (FDR) < 0.05 and | log_2_ (fold change) |≥ 1 (Varet et al. 2016). All DEGs were tested for statistical enrichment in Gene Ontology (GO) terms and Kyoto Encyclopedia of Genes and Genomes (KEGG) pathways using the KOBAS 3.0 software (http://kobas.cbi.pku.edu.cn/index.php) with corrected *p*value < 0.05 (Li et al. 2016; Si et al. 2020). PCA plot of genes identified by RNA-Seq of OM31, IM31 and WT (Fig. S1).

### Subcellular localization

The full length CDS sequence of *GhMPK31* was cloned into the green fluorescent protein (GFP) vector pGWB405 via C-terminal fusion. The recombinant vectors pGWB405-GhMPK31 were transiently expressed in tobacco epidermal cells following *Agrobacterium*-mediated transfection. The localization of the protein was observed using Olympus FV1200 confocal microscope after 2 days of *Agrobacterium* transfection. CBL1: RFP was used as a plasma membrane marker, HY5: RFP was used as a nucleus marker.

### Yeast two-hybrid, LCI and BiFC assays

To characterize the interaction between GhMPK31 and GhRBOHB proteins, the CDS of *GhMPK31* was cloned into pGBKT7 using as bait vector and transformed into yeast strain Y2H. Then the CDS of *GhRBOHB* were constructed in pGADT7 as prey vectors and transformed into yeast strain Y187. The interactions between bait and prey were detected by the growth on SD -Leu-Trp (SD-2) medium and SD-Leu-Trp-His-Ade (SD-4) medium, respectively.

For the Luciferase Complementation Imaging (LCI) assays, the CDSs of *GhMPK31* and *GhRBOHB* were cloned into the JW771 and JW772 vectors, respectively. The recombinant vectors were transformed into *Agrobacterium tumefaciens* GV3101 and transiently expressed in *Nicotiana benthamiana* leaves by needleless syringes. Fluorescence signals of LUC luminescence in LCI was observed by cryogenically cooled CCD camera (NightSHADE LB985). For Bimolecular Fluorescence Complementation (BiFC) assays, the CDSs of *GhMPK31* and *GhRBOHB* were respectively constructed to the vector pxy104-cYFP and pxy106-nYFP. The vectors were transformed into *A. tumefaciens* strain GV3101 and transiently expressed in *N. benthamiana* leaves via needleless syringes. The fluorescence in the *N. benthamiana* epidermal cells was observed 60 h later using a confocal microscope (Olympus FV1200).

### qRT-PCR

For each sample, 3 μg of RNA was reverse transcribed into cDNA using M-MLV reverse transcriptase (Promega). The Real-time quantitative PCR (RT-qPCR) reactions were performed using the QuantStudio 6 Flex Real-Time PCR System (Applied Biosystems), and *GhUB7* was used as an internal control for other genes.

### DAB staining and H_2_O_2_ measurement

H_2_O_2_ was detected by an endogenous peroxidase-dependent in situ histochemical staining procedure using 3, 3ʹ-diaminobenzidine tetrahydrochloride (DAB). After the detached leaves were thoroughly immersed in DAB staining solution, a vacuum was applied for 15 min to facilitate entry of the staining solution into the intercellular space. After an 8 h soaked in darkness, it was exposed to light for 1 h. The remaining DAB staining solution on the leaves was rinsed with distilled water. Subsequently, the leaves were immersed multiple times in a decolorization solution (75% ethanol + 5% glycerin) under dark conditions at 37 °C until chlorophyll was completely removed. The presence of H_2_O_2_ production was evidenced by the formation of a reddish-brown precipitate in the transparent leaves. The concentration of H_2_O_2_ from leaves was determined using a H_2_O_2_ quantification kit (#C500069, Sangon Biotech, Shanghai, China).

### Quasi-targeted metabolomics data acquisition

Tissues (100 mg) were individually grounded with liquid nitrogen and the homogenate was resuspended with prechilled 80% methanol by well vortex. Subsequently, the samples were incubated on ice for 5 min and centrifuged at 15,000 g, 4 °C for 20 min. A portion of the supernatant was diluted to a final concentration containing 53% methanol using LC–MS grade water. Next, the samples were transferred to a fresh Eppendorf tube and centrifuged again at 15,000 g, 4 °C for 20 min. Lastly, the supernatant was injected into the LC–MS/MS system for analysis (Want et al. 2013).

The detection of the experimental samples utilized MRM (Multiple Reaction Monitoring) and relied on the Novogene in-house database. Q3 was used for metabolite quantification. Q1, Q3, RT (retention time), DP (declustering potential) and CE (collision energy) were used for metabolite identification. The data files generated by HPLC–MS/MS were processed using SCIEX OS Version 1.4 for peak integration and correction. The main parameters were set as follows: minimum peak height (500), signal/noise ratio (5), and Gaussian smooth width (1). The area of each peak represents the relative content of the respective substance.

### Quasi-targeted metabolomics data analysis

These metabolites were annotated using the KEGG database (http://www.genome.jp/kegg/), HMDB database (http://www.hmdb.ca/) and Lipidmaps database (http://www.lipid maps.org/). Principal components analysis (PCA) and Partial least squares discriminant analysis (PLS-DA) were performed using the metaX software (Luo et al. 2015) (Fig. S2). We applied univariate analysis (*t*-test) to calculate the statistical significance (*p*-value). Metabolites with VIP > 1 and *p*-value < 0.05, and fold change (FC) ≥ 2 or FC ≤ 0.5 were considered as differential metabolites. Volcano plots were used to identify metabolites of interest based on the Log2 (FC) and − log10 (*p*-value) of metabolites using the ggplot2 library in R language. Heat maps were generated using z-scores to normalize the intensity areas of differential metabolites and plotted using the Pheatmap package in R. The correlation between differential metabolites was analyzed using cor () in R language with the "Pearson" method. The statistical significance of the correlation between differential metabolites was then calculated using the cor.mtest () in R language. *p*-value < 0.05 was considered statistically significant. Correlation plots were generated using the corrplot package in R language. The functions of these metabolites and metabolic pathways were investigated by utilizing the KEGG database. Enrichment analysis of metabolic pathways for differential metabolites was conducted. Metabolic pathways were considered enriched when the ratio were satisfied by *x*/*n* > *y*/*N*, metabolic pathway was considered statistically significantly enriched when its *p*-value < 0.05.

### Selective and non-selective feeding assays of *H. armigera and S. litura*

Both the T1 generation transgenic materials and the WT materials were cultivated under identical field conditions. Two types of leaves were selected from the OM31 lines: leaves without necrotic blotches and leaves showing necrotic blotches. Similarly, leaves from the corresponding parts of WT plants were selected as contrast. Choice feeding assays were performed in plastic boxes measuring 29 × 44 cm. First, we starved the third instar larvae for 6 h, then we symmetrically and evenly arranged the removed leaves. 12 starved larvae were placed in the middle of each plastic box and allowed to feed freely for 24 h. The consumption of cotton leaves was calculated by ImageJ software. For the non-selective feeding experiment with the moth, 9 cm round glass petri dishes were used to hold leaves from WT plants and OM31 separately. Second-instar *S. litura* larvae with comparable body weight were chosen and placed individually on the leaves, with one larva per dish. The body weight of the larvae was recorded at day 0 and day 5 (Hu et al. 2018).

## Results

### Comprehensive analysis of *MAPK* gene family in cotton

**Fig. 1:**
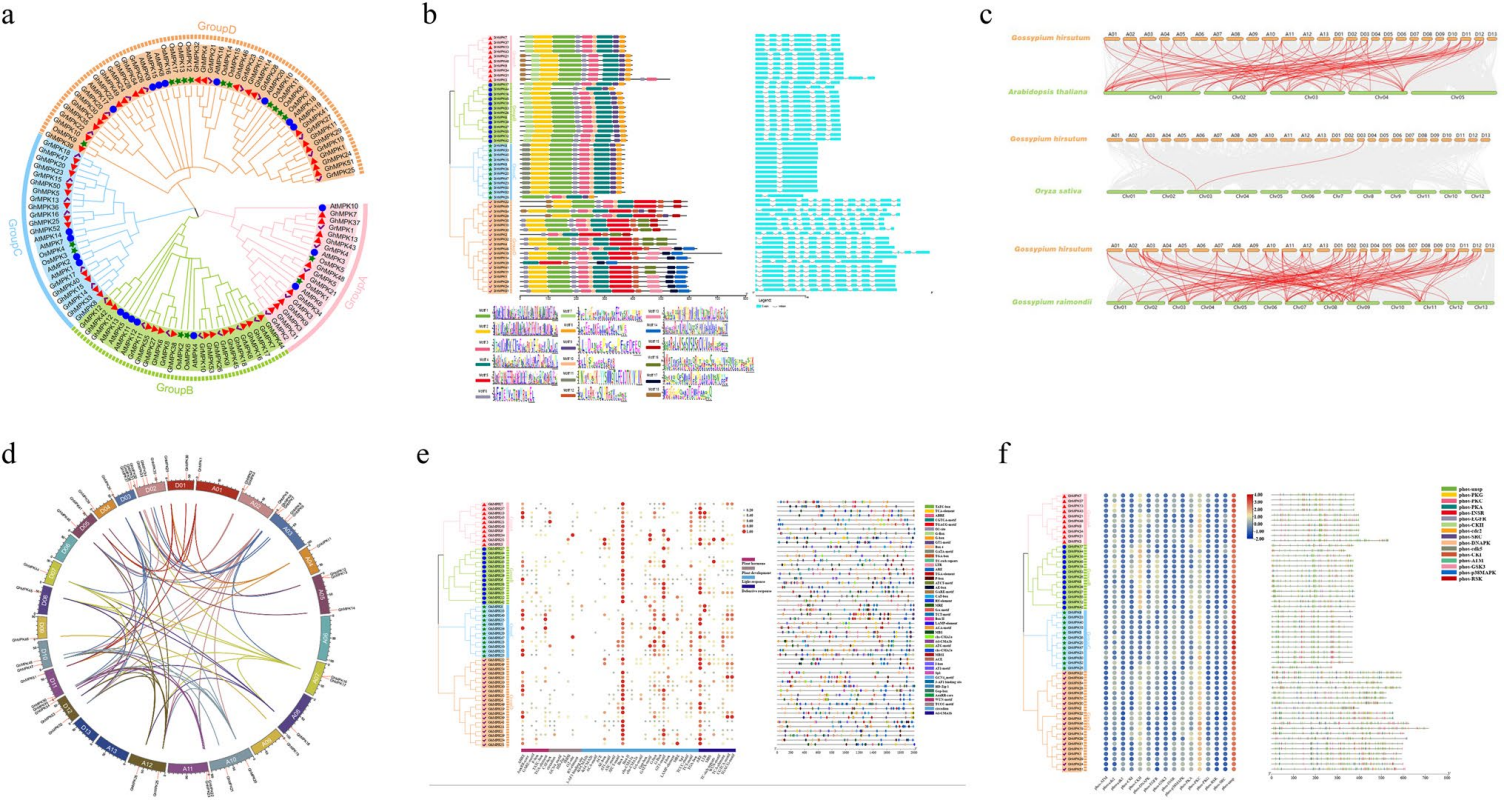
Comprehensive analysis of *MAPK* gene family in *Gossypium hirsutum*. **a** Phylogenetic analysis of *MAPK* gene families in *G. hirsutum*, *A. thaliana*, *O. sativa* and *G. raimondii*. A, B, C and D indicate different gene clusters (groups). The different shapes and colors of the symbols indicate different species. **b** Phylogenetic relationships, conserved protein motifs and gene structures of *GhMPKs*. On the left is the phylogenetic tree of the *MAPK* gene family in *G. hirsutum*. In the middle is the motif distribution of the MAPK protein. There are 18 motifs in total, displayed in different colored boxes, below is the sequence logo for MAPK proteins motifs. On the right is the exon–intron structure of the *GhMPKs* gene. The blue box represents the exon and the black line represents the intron. **c** Collinearity analysis of *MAPK* genes between *GhMPKs* and three other plants. Grey lines indicate collinear blocks within the *G. hirsutum* genome and other plant genomes, and the red curve indicates *MAPK* genes with collinearity. **d** Collinearity analysis and chromosomal location of the *GhMPK* gene family. The chromosome of *G. hirsutum* was distinguished by different color. Each *GhMPK* gene is marked with a short red line on the chromosome and collinear gene pairs are represented by a color curve. **e** *Cis*-acting elements in the promoter regions of *GhMPKs*. On the left is the phylogenetic tree of the *MAPK* gene family in *G. hirsutum*. In the middle is a heat map of the number of all *cis*-acting elements. All *cis*-elements are categorized by function into four categories, *cis*-elements with similar functions are displayed in the same color. On the right is the promoter acting element of the *GhMPK* genes. The black line indicates the promoter length of the *GhMPK* genes. The different colored boxes on the right represent *cis*-acting elements with different functions. **f** Prediction of phosphorylation sites of *GhMPKs*. On the left is the phylogenetic tree of the *MAPK* gene family in *G. hirsutum*. In the middle is a heat map of the number of all phosphorylation sites. On the right is the distribution of protein kinase phosphorylation sites in *GhMPKs* (colour figure online)

To comprehend the significance of the *MAPK* gene family in upland cotton, both BLASTP and Hidden Markov Model (HMM) searches against *G. hirsutum* (NBI and HAU) protein databases were conducted using *A. thaliana*, *O. sativa* and *G. raimondii* MAPK proteins as query sequences. After validation of the conserved domains, we successfully identified and characterized 55 putative *GhMPK* genes (Table S1). These putative *GhMPK* genes encoded proteins that varied in length from 275 to 716 amino acids, the proteins Mw ranging from 31.62 to 82.45 kDa, and the PI of the proteins fell within the range of 4.81 to 9.72. The subcellular localization prediction indicated that the majority of GhMPK proteins were located in the cytoplasm and nucleus. However, with the exception of GhMPK19, most MAPK proteins lack a signaling transmembrane domain, while GhMPK19 is the only one possessing two transmembrane domains. To investigate the evolutionary relationships of *GhMPKs*, we constructed a phylogenetic tree based on amino acid sequences of 55 *GhMPKs* from *G. hirsutum*, 20 *AtMPKs* from *A. thaliana*, 17 *OsMPKs* from *O. sativa* and 28 *GrMPKs* from *G. raimondii* (Fig. 1a). The findings indicated that all *MAPKs* were clustered into four groups: A, B, C and D. Notably, Group D constituted the largest cluster, featuring the highest count of *MAPKs*. In *GhMPKs* family, the TEY type of T-loop belongs to Groups A, B and C, whereas TDY type of T-loop is classified into Group D (Table S1).

Moreover, we conducted an analysis of the distribution of motifs and the exon–intron structure of GhMPK proteins (Fig. 1b). A total of 18 conserved motifs were detected, which motifs 1, 2, 3, 4 and 6 appearing in nearly all GhMPK proteins, suggesting that GhMPKs were highly conserved. However, different groups contained its certain unique motifs. Motif 7 was found exclusively in groups A and B, whereas motif 11 was specific to group C. Additionally, group D contained unique motifs 5, 13, 14, 15, 16 and 17. These findings indicate that GhMPKs in different subgroups may possess distinct functions. The results of exon intron structure analysis showed that the gene structures of *GhMPK* within the same group exhibited a higher degree of conservation. For example, each member of group C contained 2 exons and 1 intron, and all *GhMPKs* of group B had the same intron–exon distribution pattern. However, group D exhibited a significantly higher number of exons and introns compared to other groups, with varied distribution patterns. These findings suggest that group D GhMPKs potentially demonstrates more complex functions. Chromosome mapping analysis revealed an uneven distribution of 47 *GhMPKs* across 22 out of 26 *G. hirsutum* chromosomes, with the remaining eight members distributed on the scaffold. Noticeably, *GhMPKs* were absent on chromosomes A06, D06, A13 and D13 (Fig. 1d). Additionally, our findings revealed that both subgroup A and subgroup D contained 23 and 24 members respectively, indicating an equal distribution of members within the *GhMPKs* family across these subgroups. By collinear analysis of the *GhMPK* genes family, 75 collinear pairs were identified on 22 chromosomes (Fig. 1d). The majority of the genes exhibited 1–2 collinear pairs, while a few had 4. In addition, the collinearity of *G. hirsutum* with *A. thaliana*, *O. sativa* and *G. raimondii* for *MAPK* was investigated and identified 77, 2 and 114 gene pairs, respectively (Fig. 1c). Homologous gene pairs demonstrate significant differences across species, suggesting a stronger evolutionary bias of *GhMPK* towards dicots (*A. thaliana and G. raimondii*) compared to monocots (*O. sativa*), thereby elucidating the underlying evolutionary mechanism of the *GhMPK* family in various species.

To further investigate the function of *GhMPKs*, the cis-acting elements in the promoter region of *GhMPKs* were identified and analyzed, which can be classified into four categories associated with plant hormones, plant development, photo response and defense/stress response (Fig. 1e). Among them, nearly all *GhMPK* genes contained elements related to light response, followed by stress response elements, with the least number of elements associated with the development of plants. These findings demonstrate that *GhMPKs* could potentially contribute to multiple pathways involved in plant growth and development. We also predicted a total of 2147 reliable phosphorylation sites across *GhMPK* gene family, with the number of phosphorylation sites for each member ranging from 22 to 67 (Fig. 1f). *GhMPK49* exhibited the highest number of phosphorylated sites (67), whereas *GhMPK8* contained the lowest number of phosphorylated sites (22). Nevertheless, we observed no positive correlation between the number of phosphorylation sites and the length of the amino acid sequence. As a crucial protein kinase class, the activation of MAPK typically occurs through phosphorylation. Therefore, analyzing phosphorylation sites plays a vital role in comprehending the mechanisms underlying GhMPK proteins.

### Expression patterns of *GhMPK* genes under various abiotic and biotic stresses

**Fig. 2:**
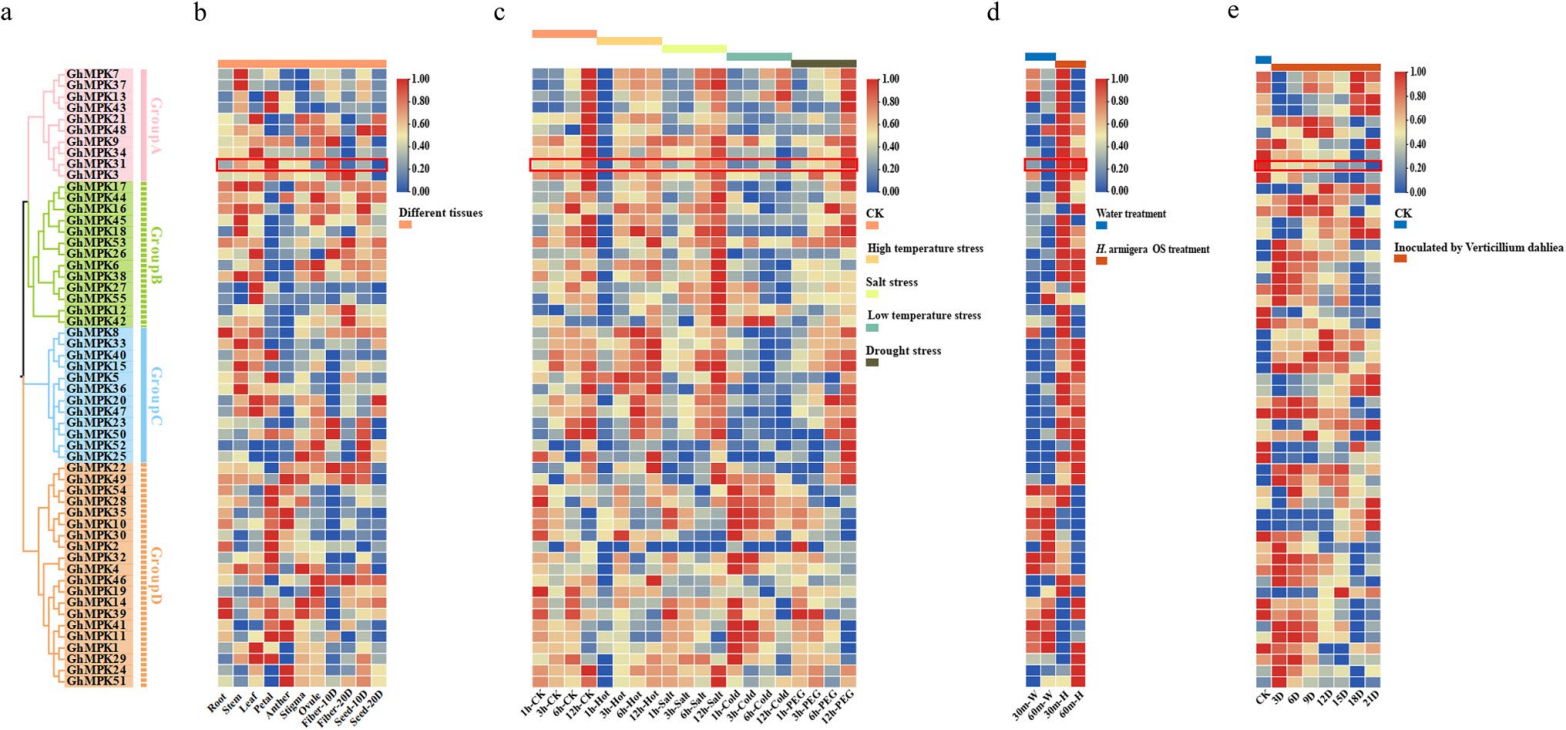
Expression patterns of the *MAPK* gene family under different tissues and stress conditions. **a** Phylogenetic tree of the *MAPK* gene family in *G. hirsutum*. Different colors represent different subgroups. **b** Analysis of expression profiling of *GhMPKs* in different tissues. From left to right are root, stem, leaf, petal, anther, stigma, ovule, fiber 10 days, fiber 20 days, seed 10 days and seed 20 days. Red indicates increased expression, blue indicates decreased expression. **c** Abiotic stress-induced expression profiles of *GhMPKs* under heat, cold, PEG and salt treatments. Red indicates increased expression, blue indicates decreased expression. **d** Expression patterns of *GhMPK* genes induced by *H. armigera* OS. Red indicates increased expression, blue indicates decreased expression. **e** Expression patterns of *GhMPK* genes induced by *V. dahliae*. Red indicates increased expression, blue indicates decreased expression (colour figure online)

Extensive research has demonstrated a close relationship between gene expression and functionality. By using the protein sequence of GhMPKs to construct a phylogenetic tree, which revealed that all GhMPKs cluster into four distinct subgroups (Fig. 2a). By analyzing the expression of *GhMPKs* between different subgroups under various stresses, a better exploration of the potential role of the *MAPK* gene family in upland cotton can be conducted. Initially, the expression of *GhMPK* genes were examined across various cotton tissues. Analysis of tissue expression patterns revealed widespread expression of all members of the *GhMPKs* in nearly all tested tissues. However, there were significant variations in their transcription levels, indicating functional distinctions within this gene family during growth and development (Fig. 2b). Afterwards, the expression patterns of *GhMPKs* were investigated in cotton leaves when subjected to four different abiotic stress conditions, namely heat, salt, cold and drought (Fig. 2c). Following high temperature (38 ℃) treatment, the expression of 96% *GhMPKs* in the experimental group was significantly down-regulated after 1 h. However, after 3 h, the expression levels of almost all *GhMPKs* were upregulated, with 31 genes showing higher expression levels than the control group, only *GhMPK11* and *GhMPK25* exhibited continued decline. After 1 h of salt (400 mM NaCl) and drought (20% PEG) treatment, 41 *GhMPKs* exhibited similar expression patterns, and compared with the control, *GhMPK28* and *GhMPK30* were significantly down-regulated under both treatments. After 1 h of low temperature (4 °C) treatment, the upregulated *GhMPKs* were mainly concentrated in subgroup D compared with the control, while most of the genes in subgroup A and C exhibited downregulation. Both *GhMPK10* and *GhMPK35* exhibited significant upregulation following exposure to cold and salt treatments, while displaying significant downregulation after heat treatment. These findings indicate that the *GhMPK* gene family experiences significant induction or inhibition in response to diverse abiotic stresses.

Next, the transcriptome of this gene family were analyzed at different time points after treatment with *H. armigera* OS and *V. dahliae* infestation (Fig. 2d, e). The results revealed that 75.6% of *GhMPKs* exhibited up-regulation after 30 min of treatment with *H. armigera* OS, in comparison to the control group. Additionally, it was observed that 14 *GhMPK* genes displayed down-regulation, with 12 genes belonging to subgroup D, while all the genes in subgroup C exhibited up-regulation. *GhMPK31* and *GhMPK40* exhibited significant up-regulation in transcription upon treatment with *H. armigera* OS, showcasing high expression levels. Specifically, their transcription levels were up-regulated by more than 1.5-fold at both the 30-min and 60-min time points. After 3 days of *V. dahliae* inoculation, transcription levels of 53% *GhMPKs* genes increased compared with the control. *GhMPK15* and *GhMPK51* transcription levels were up-regulated by more than 1.5 times maintaining the high expression levels. The transcription of *GhMPK14* and *GhMPK39* was consistently inhibited for a period of 21 days following inoculation. These results indicate that the *GhMPK* gene family also plays an important role in response to biological stress.

### Overexpression of *GhMPK31* resulted in hypersensitive response (HR)-like cell death and dwarfing of cotton

*GhMPK31* exhibits significant induction or inhibition in response to various biotic and abiotic stresses, particularly showing high expression levels under the treatment of *H. armigera* OS. Additionally, it shares homology with *AtMPK6* (Yuasa et al. 2001), one of the extensively studied genes in plants. Consequently, we made the decision to conduct further research into the functionality of *GhMPK31*.

**Fig. 3:**
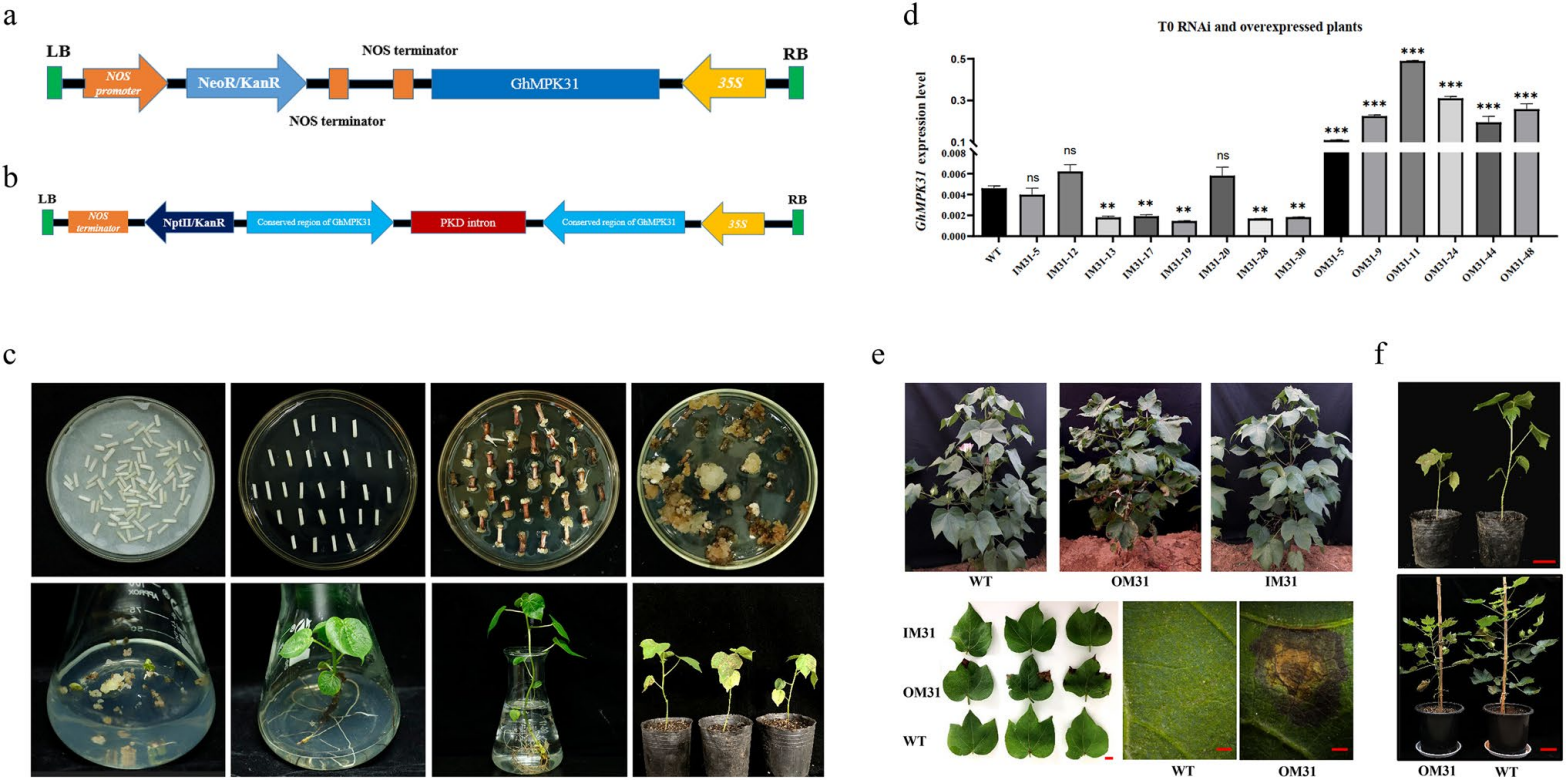
Creation of *GhMPK31* transgenic plants with overexpression and RNAi. **a** Construction of overexpression *GhMPK31* vector. **b** Interference *GhMPK31* vector construction. **c** Genetic transformation process of cotton. **d** Determination of *GhMPK31* expression level in transgenic materials. The expression of *GhUB7* was used as internal control. Means ± SE (*n* = 3). Significant difference analysis was done by Student’s *t*-tests (**, *p* < 0.01, ***, *p* < 0.001). **e** Phenotypes comparison between T1 transgenic materials and WT plants. Compared with WT plants, OM31 lines showed necrotic lesions in the leaves, a kind of HR-like cell death phenotype. The red line represents 1 cm. **f** Comparison of plant height between T1 generation OM31 and WT plants. The red line represents 10 cm (colour figure online)

To determine the functional role of *GhMPK31* in cotton, overexpression and RNAi vectors were constructed based on the CDS of *GhMPK31*, followed by the *Agrobacterium*-mediated genetic transformation, resulting in the generation of independent transgenic plants (Fig. 3a, b, c). Two independent RNAi lines (IM31-19 and IM31-28) and two independent overexpression lines (OM31-11 and OM31-24) were identified based on the expression level of *GhMPK31* for subsequent studies (Fig. 3d). Phenotypic analysis of the T1 generation OM31 lines showed the presence of necrotic lesions on leaves and a dwarf plant architecture, whereas the T1 generation RNAi lines exhibited no discernible differences compared to the WT (Fig. 3e, f). We observed that necrotic blotches on the leaves of OM31 lines appeared during the seedling stag persisted throughout the entire growth period of cotton plants. Necrotic blotches were absent on the new leaves, but rather, they were typically found on the older leaves. Thus, these results suggest that the necrotizing spots are not caused by viruses but by a phenomenon known as HR-like cell death.

### RNA-seq analysis revealed dramatic gene expression difference among OM31, IM31 and WT cotton plants

**Fig. 4:**
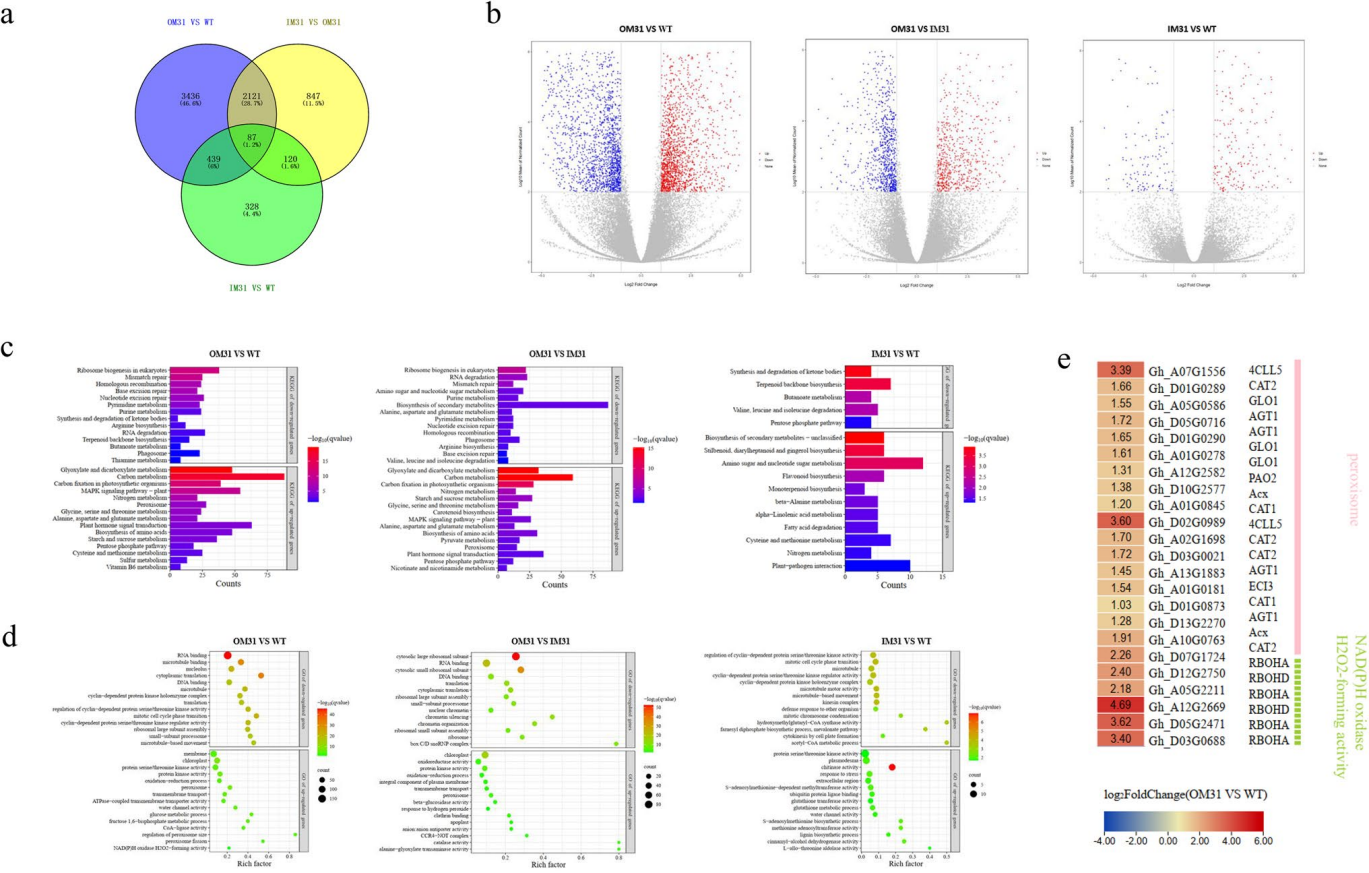
RNA-seq analysis revealed the effect of *GhMPK31* on gene expression. **a** Venn diagram showing overlap of DEGs between three lines. **b** Differential gene volcano map of three groups. Red represents up-regulated DEGs, and blue represents down-regulated DEGs. (*q*value < 0.05 and | log2 FC|≥ 1). **c** Three groups of KEGG pathway enrichment up-regulated or down-regulated DEGs. (*q*value < 0.05). **d** Three groups of up-regulated DEGs or down-regulated DEGs enrichment top 15 GO terms (*q*value < 0.05). **e** The differentially expressed ROS-related genes were identified by RNA-seq method. The color in each cell represents the value of the log_2_ fold change (OM31 VS WT) (colour figure online)

We investigated the expression differences among T1 generation OM31, IM31 and WT cotton plants using RNA-seq. A total of 6038 DEGs were identified in the OM31 lines compared with the WT cotton plants, of which 2844 DEGs were down-regulated and 3239 DEGs were up-regulated. A total of 3175 DEGs were identified in the comparison between OM31 and IM31 plants. In contrast, only 974 DEGs were identified in the comparison between IM31 and WT plants (*p*-adjust value < 0.05 and | log_2_ (fold change) |≥ 1) (Fig. 4a, b), suggesting that interference with *GhMPK31* has no significant effect on the expression of other genes. GO term enrichment analysis and KEGG pathway enrichment analysis were separately conducted for the three groups: OM31 vs WT, OM31 vs IM31 and IM31 vs WT, based on the up-regulated and down-regulated DEGs (Fig. 4c, d). Interestingly, the top 15 enriched KEGG pathways in both the OM31 vs WT and OM31 vs IM31 groups exhibited up-regulation of the same pathways. These pathways encompassed Carbon fixation in photosynthetic organisms, Carbon metabolism, Nitrogen metabolism and the Peroxisome pathway. Moreover, the identical GO term was identified within the top 15 up-regulated GO terms in both groups, encompassing chloroplast, peroxisome, NADPH oxidase and H_2_O_2_-forming activity. In addition, we found that some key genes (*RBOHA*, *RBOHD*, *CAT1* and *CAT2*) associated with H_2_O_2_ were significantly induced in OM31 plants (Fig. 4e). These results demonstrated that overexpression of *GhMPK31* in plants may be related to processes such as carbon fixation, nitrogen metabolism and H_2_O_2_ generation and decomposition.

### H_2_O_2_ levels increase in ***GhMPK31***-overexpression plants

**Fig. 5:**
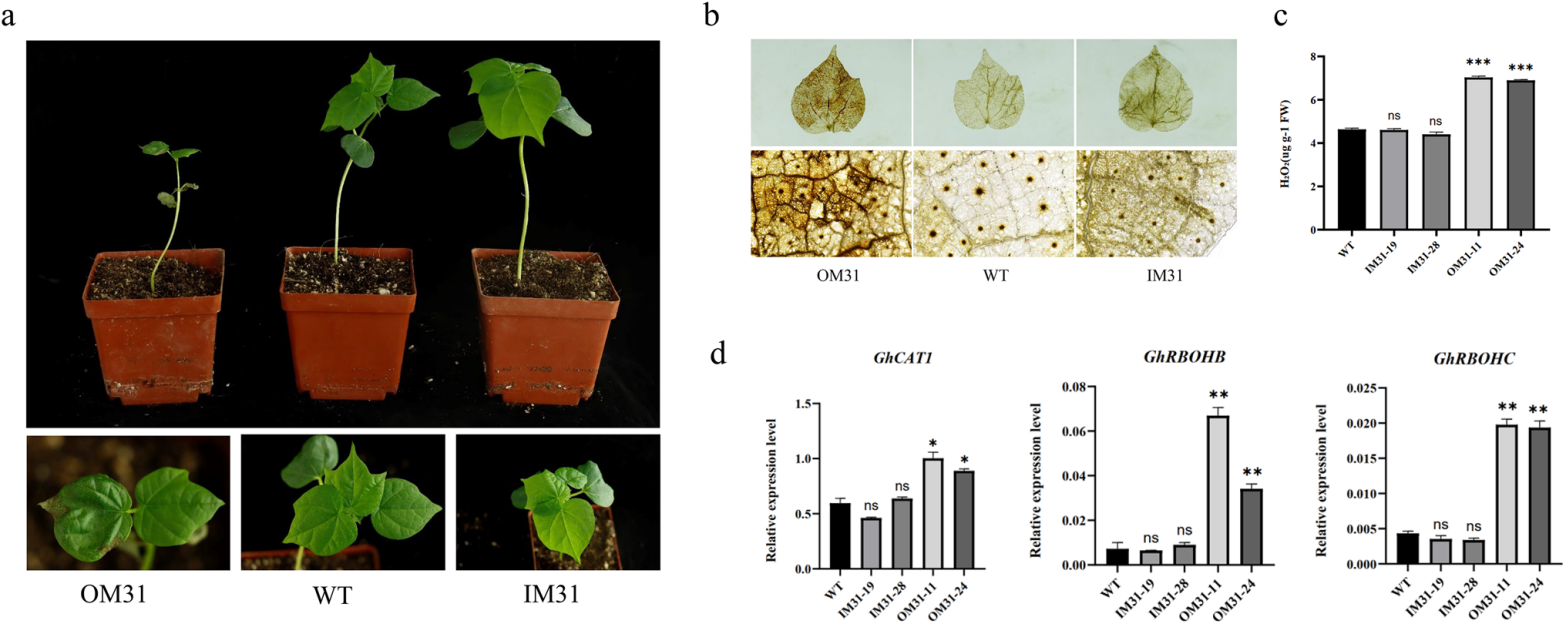
Overexpression of *GhMPK31* leads to accumulation of H_2_O_2_ in plants. **a** T2 generation OM31, IM31 and WT. **b** Presence of H_2_O_2_ in cotton leaves visualized by staining with DAB. Oxidized DAB formed a reddish-brown deposition. **c** H_2_O_2_ concentration in OM31, IM31 and WT leaves. Error bars represent ± standard errors of three biological replicates. Means ± SE (*n* = 3). **d** The relative expression of H_2_O_2_ related genes is in OM31, IM31 and WT backgrounds. The expression of *GhUB7* was used as internal control. Means ± SE (*n* = 3). Statistical analyses were performed using Student’s t test. *, *p* < 0.05; **, *p* < 0.01. ***; *p* < 0.001. All of the experiments were repeated at least three times with similar results

Transcriptome results suggest that overexpression of *GhMPK31* leads to the upregulation of genes related to H_2_O_2_ and the phenomenon of HR-like cell death in plants is often associated with the excessive accumulation of H_2_O_2_ (Liu et al.^2007^). Therefore, we hypothesize that the necrotic blotches phenotype observed in the leaves of OM31 plants is a result of the excessive accumulation of H_2_O_2_. To validate this hypothesis, the seeds of T1 generation OM31, IM31 and WT were simultaneously cultivated under identical environmental conditions (Fig. 5a). Six weeks later, DAB staining was conducted on the leaves from the corresponding parts of the three lines. The results revealed a significant accumulation of reddish-brown precipitate in the OM31 lines, however, neither IM31 lines nor WT showed any such accumulation (Fig. 5b). The H_2_O_2_ content test demonstrated significantly higher levels of H_2_O_2_ in the OM31 lines compared to the IM31 lines and WT (Fig. 5c). Additionally, using RT-qPCR, we identified several NADPH oxidase and catalase genes involved in H_2_O_2_ synthesis and decomposition. The results indicate significantly elevated expression levels of *GhCAT1*, *GhRBOHB* and *GhRBOHC* in the OM31 lines compared to both WT and IM31 lines (Fig. 5d). These results suggest that overexpression of *GhMPK31* leads to an elevation in H_2_O_2_ content in cotton leaves, which might lead to HR-like cell death and dwarfing in OM31 plants.

### *GhMPK31* physically interacts with *GhRBOHB* revealed by Y2H, BiFC and LCI

**Fig. 6:**
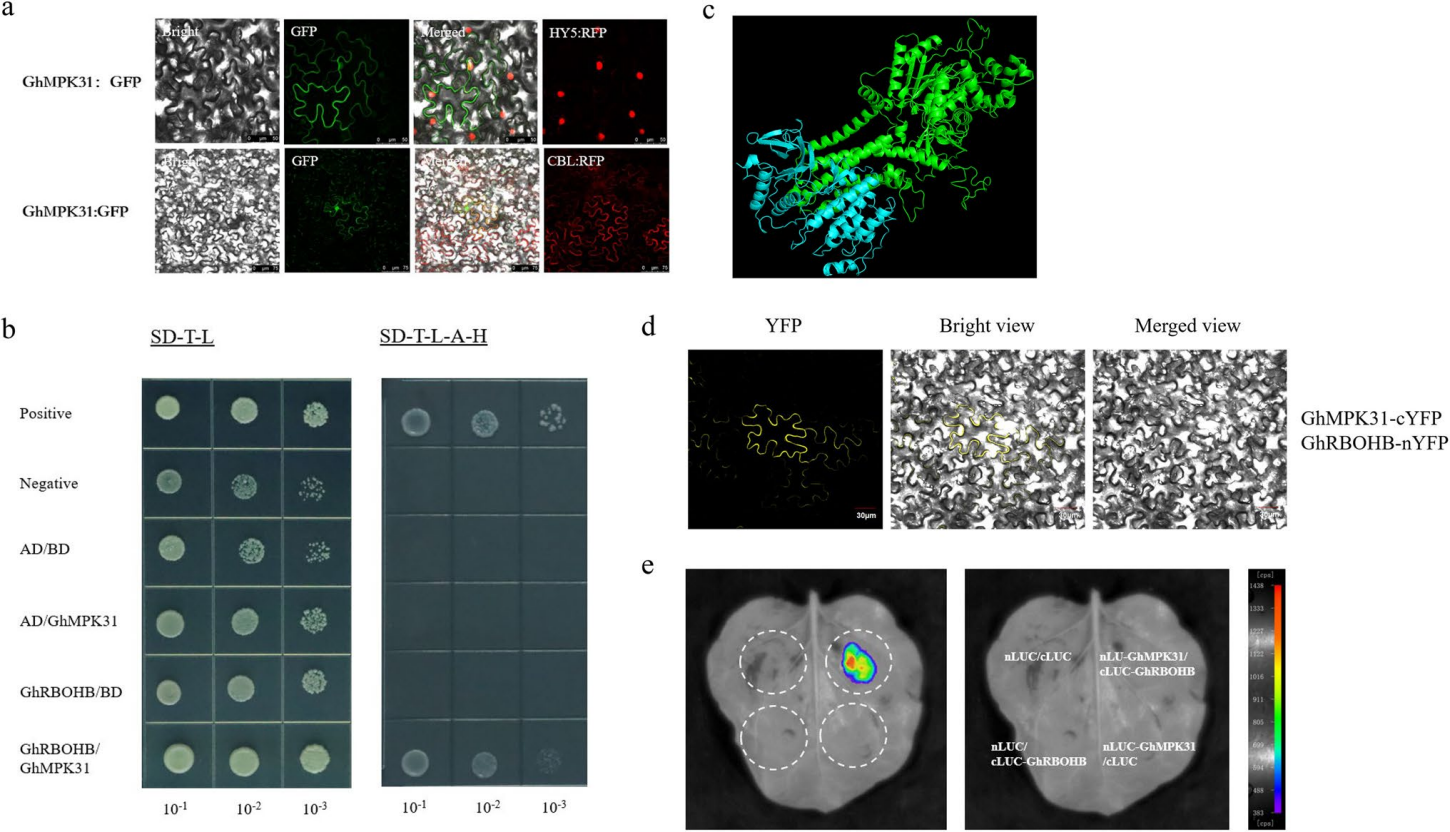
GhMPK31 physically interacts with GhRBOHB in cotton. **a** The subcellular localization of GhMPK31: GFP protein. GFP fluorescence was observed after transiently expressing GhMPK31: GFP protein in tobacco epidermal cells. CBL and HY5 were used as plasma membrane and nucleus markers, respectively. **b** Y2H assay for GhMPK31- GhRBOHB interaction. SD-2 (-Trp/-Leu), SD-4 (-Trp/-Leu/-His/-Ade). **c** 3D protein structural model of GhMPK31 and GhRBOHB interaction. **d** BiFC assay between GhMPK31-cYFP and GhRBOHB-nYFP in tobacco epidermal cells. Bars = 30 μm. **e** LCI analysis of GhMPK31-nLUC and GhRBOHB-cLUC in tobacco leaves

To further explore the mechanism of *GhMPK31* regulating ROS, we firstly investigated the subcellular localization of *GhMPK31*. The full CDS length of *GhMPK31* was fused to the N-terminus of GFP and transiently expressed in tobacco epidermis, and through confocal spectral microscope fluorescence signals were observed in both cell membrane and nucleus (Fig. 6a). Then, we carried out a yeast two-hybrid (Y2H) assay to screen potential interacting proteins and found the direct interaction of GhMPK31 protein with the NADPH oxidase (GhRBOHB) (Fig. 6b). We used Y2H-AOS protein interaction prediction analysis to construct the three-dimensional structure of the interaction model of these two proteins, and the protein interaction confidence score was 9.6, which proved that this interaction was highly possible (Fig. 6c). The interaction between *GhMPK31* and *GhRBOHB* was further confirmed using the BiFC assay and LCI assay (Fig. 6d, e). These results revealed that GhMPK31 interacts with GhRBOHB in cotton.

### Overexpression of *GhMPK31* reduces the accumulation of cotton defense metabolites and results in sensitive against herbivorous insects’ infestation

**Fig. 7:**
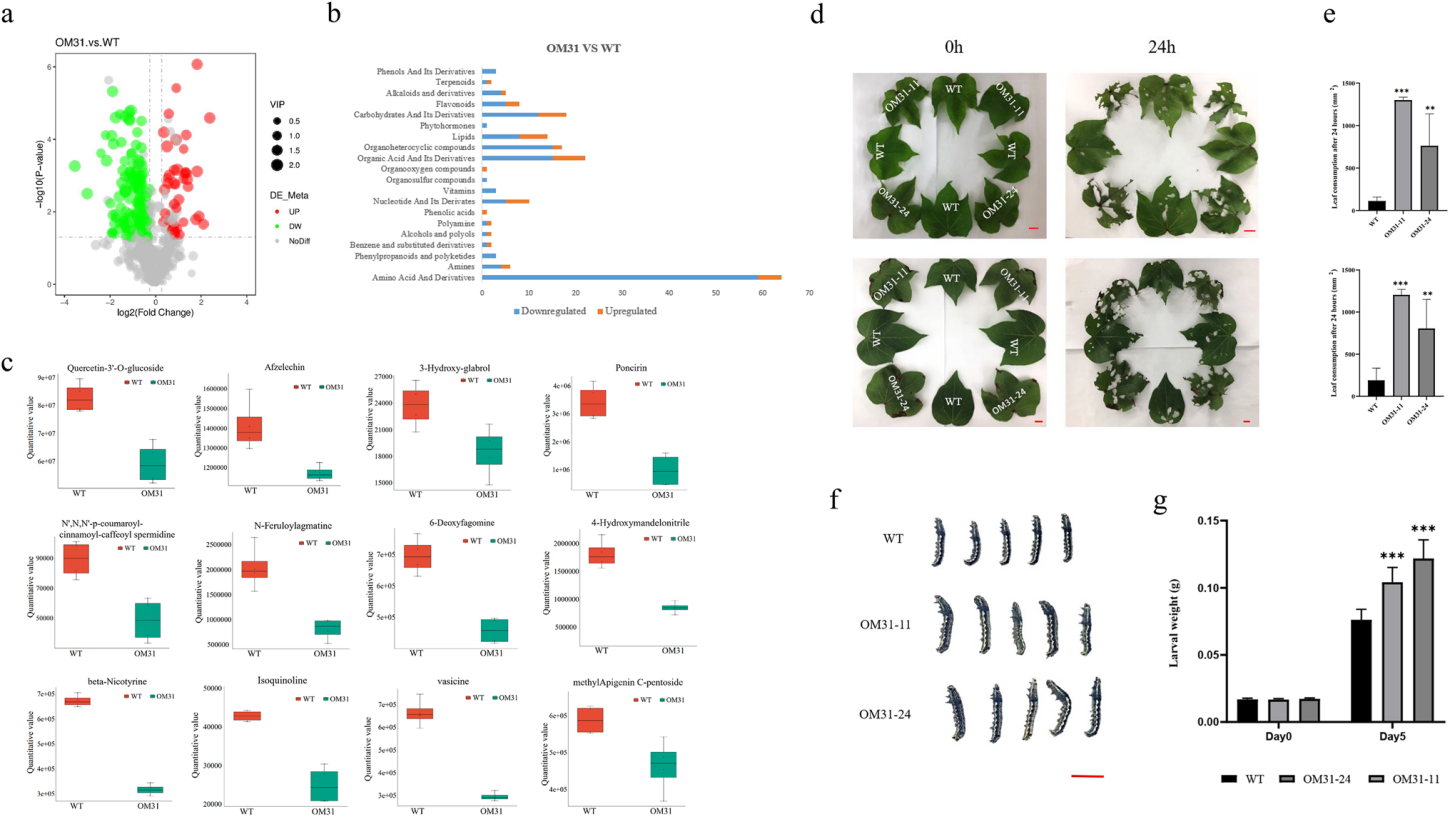
Overexpression of *GhMPK31* reduced the accumulation of defensive metabolites and decreased the defense ability of cotton against herbivorous insects. **a** Volcanic map of differential metabolites between OM31 and WT. Red represents up-regulated metabolites and green represents down-regulated metabolites, (*q*value < 0.05). **b** Number of differential metabolites in each compound class in OM31 mutants. Orange represents up-regulated metabolites and green represents down-regulated metabolites (*q*value < 0.05). **c** Box diagram of defense-related metabolites between OM31 and WT. Green for WT, red for OM31. Error bars represent ± standard errors of four replicates. Means ± SE (*n* = 4). **d** Choice feeding assay of *H. armigera*. The third-instar *H. armigera* were pre-starved for 6 h and photographed at 0 h and 24 h. The red line represents 1 cm. **e** Leaf consumption in the choice feeding assay for *H. armigera*. Means ± SE (*n* = 6). **f** Comparison between the body-size of larvae fed on WT plants and OM31 lines leaves after 5 days of non-selective feeding. The red line represents 1 cm. **g** Average larval weight on days 0 and 5. Means ± SE (*n* = 12). Statistical analyses were performed using Student’s *t* test. *, *p* < 0.05; **, *p* < 0.01. ***; *p* < 0.001. All of the experiments were repeated at least three times with similar results

Secondary metabolites are widely recognized as key players in the plant's response to pest stress. Quasi-targeted metabolomics experiments were performed on T1 generation OM31 and WT plants to identify the variations in defense-related metabolites between them, and to investigate the impact of *GhMPK31* on cotton's defense against herbivorous insects. A total of 698 metabolites were detected, with 185 of them exhibiting significant differences (*p* < 0.05). In comparison to WT plants, 76.7% of metabolites in OM31 lines were significantly decrease (Fig. 7a, S4). These differential metabolites were categorized into 21 distinct compound classes, most of which were classified as amino acids and their derivatives, carbohydrates and their derivatives, organic acids and their derivatives, organic heterocyclic compounds, etc. (Fig. S3). The known endogenous defense metabolites we focused on include flavonoids, alkaloids including their derivatives, phenols and its derivatives (Erb and Kliebenstein 2020), which significantly reduced in OM31 lines (Fig. 7b, c). We also assessed the expression levels of several genes associated with flavonoid synthesis using RT-qPCR. The results revealed a significant downregulation of *GhCHS1*, *GhCHI*, *GhDFR* and *GhAN3* expression in OM31 lines compared to the WT (Fig. S5). To assess the impact of *GhMPK31* on the defense capacity of cotton against insects, we conducted selective and non-selective feeding assays using *H. armigera and S. litual*. Two types of leaves were chosen for selective feeding according to the presence of necrotic blotches: leaves without necrotic blotche and leaves with visible necrotic blotches. Leaves were selected from corresponding parts of WT plants to serve as the control group. In the selection experiment, cotton leaves from both OM31 and WT plants were evenly distributed, and third-instar bollworm larvae were given unrestricted access to feed (Fig. 7d). After 24 h, we observed a significant increase in the consumption of both types of leaves in the OM31 plant lines compared to the WT plants. (Fig. 7e). In the non-selective experiment, second *S. litual* instar larvae were fed with leaves from both WT plants and OM31 lines for 5 consecutive days. It was observed that the larvae fed on OM31 leaves exhibited significantly greater weight compared to those fed on WT leaves (Fig. 7f, g). These results collectively demonstrate that the overexpression of *GhMPK31* leads to a reduction in various metabolites, including defensive substances. Consequently, this reduction weakens the cotton plant's defense against herbivorous insects.

## Discussion

*MAPK* gene family has been widely identified in different plant species, but detailed and comprehensive analysis in the *G. hirsutum* genome is relatively scarce. The analysis of phosphorylation sites and promoter *cis*-elements of *GhMPKs* provides valuable reference for functional analysis of *MAPK* gene family in upland cotton. In addition, *GhMPK31*, which is a homolog of *AtMPK6*, was screened out through the analysis of expression profiles under various stress conditions, warranting further investigation. *AtMPK6* and its homologues in other plants have been extensively studied and are considered one of the earliest genes in the *MAPK* gene family (Doczi and Bogre 2018; Xu and Zhang 2015). This gene is characterized by its important functions in plants’ life cycle, such as growth and development, biotic and abiotic stresses and hormone signaling (Jia et al. 2016; Li et al. 2018; Majeed et al. 2023; Sun et al. 2018). Research on cotton has mainly focused on its response to ABA induction, participation in drought stress and regulation of fiber development (Chen et al. 2020b; Li et al. 2017; Luo et al. 2011). However, there have been no reported studies on the regulation of cotton leaf HR-like cell death and defense capabilities.

We overexpressed *GhMPK31* in cotton, which can induce HR-like cell death in cotton leaves and dwarfing of cotton plants. This is typically associated with the outbreak of reactive ROS (Liu et al. 2007). ROS is an important second messenger in plants, which can be continuously produced in the cell through the respiration of mitochondria, chloroplasts and peroxisomes, including the superoxide anion (O_2_^−^), H_2_O_2_ and the hydroxyl radical (^.^OH). Meanwhile, it can also be eliminated by some scavenging enzymes, such as superoxide dismutase (SOD), ascorbate peroxidase (APX), glutathione peroxidase (GPX) and CAT (Apel and Hirt 2004; Lamb and Dixon 1997). Maintaining a dynamic balance of reactive ROS is crucial for plant development. ROS serves as a crucial signaling molecule, mediating plant growth, development and responses to environmental stress. However, excessive levels of ROS can induce oxidative damage to cellular membranes, proteins, carbohydrates and DNA. Particularly, rapid bursts of H_2_O_2_ can trigger programmed cell death (Liu and He 2016; Moller et al. 2007). Based on our analysis of OM31 leaves, we observed an elevated H_2_O_2_ content compared to WT, so we hypothesized that *GhMPK31* may promote HR-like cell death in cotton leaves by positively regulating H_2_O_2_ synthesis. Furthermore, the results of RNA-seq and RT-qPCR revealed a significant increase in the expression levels of genes associated with H_2_O_2_ synthesis and decomposition in OM31 leaves, providing further support for this hypothesis. The H_2_O_2_ content in IM31 plants was found to be comparable to that in WT plants, and RNA-seq analysis revealed a minimal number of DEGs between IM31 and WT. We believed that upland cotton, being a tetraploid crop, has functional redundancy of *GhMPK31*.

In recent years, studies on *MAPK6* regulating ROS in different species have been successively reported, although the specific mechanisms are not yet clear. In *A. thaliana*, *AtMPK6* mediates ABA-induced *CAT1* expression and H_2_O_2_ production (Xing et al. 2008). *NTF6* regulates NADPH Oxidase-Dependent oxidative bursts in *N. benthamiana* (Asai et al. 2008), *MPK6* mediates salt-induced iron superoxide dismutase gene expression and further induces ROS production (Xing et al.^2 015^). In soybean, *GmMPK6* induces ROS generation through the transcriptional regulation of *GmRbohI1* and increases salt tolerance in soybean (Son et al. 2023). Similarly, in this study, we detected an excessive amount of H_2_O_2_ in the leaves of OM31 plants and found direct evidence of interaction between GhMPK31 and GhRBOHB. Therefore, we speculate that GhMPK31 influences the generation of ROS through its interaction with GhRBOHB, which is also the direct cause of HR-like programmed cell death in cotton leaves. Furthermore, our wide comparative metabolomics analysis revealed a significant reduction in the levels of various defensive metabolites (such as flavonoids, alkaloids and their derivatives, phenols and their derivatives) in OM31 plants compared to the WT. This might be one of the factors to reduce OM31 defense capability shown in *H. armigera* selective feeding experiment, however, the regulatory mechanism of *GhMPK31* in regulating these pathways was not clear which requires further investigation.

**Fig. 8:**
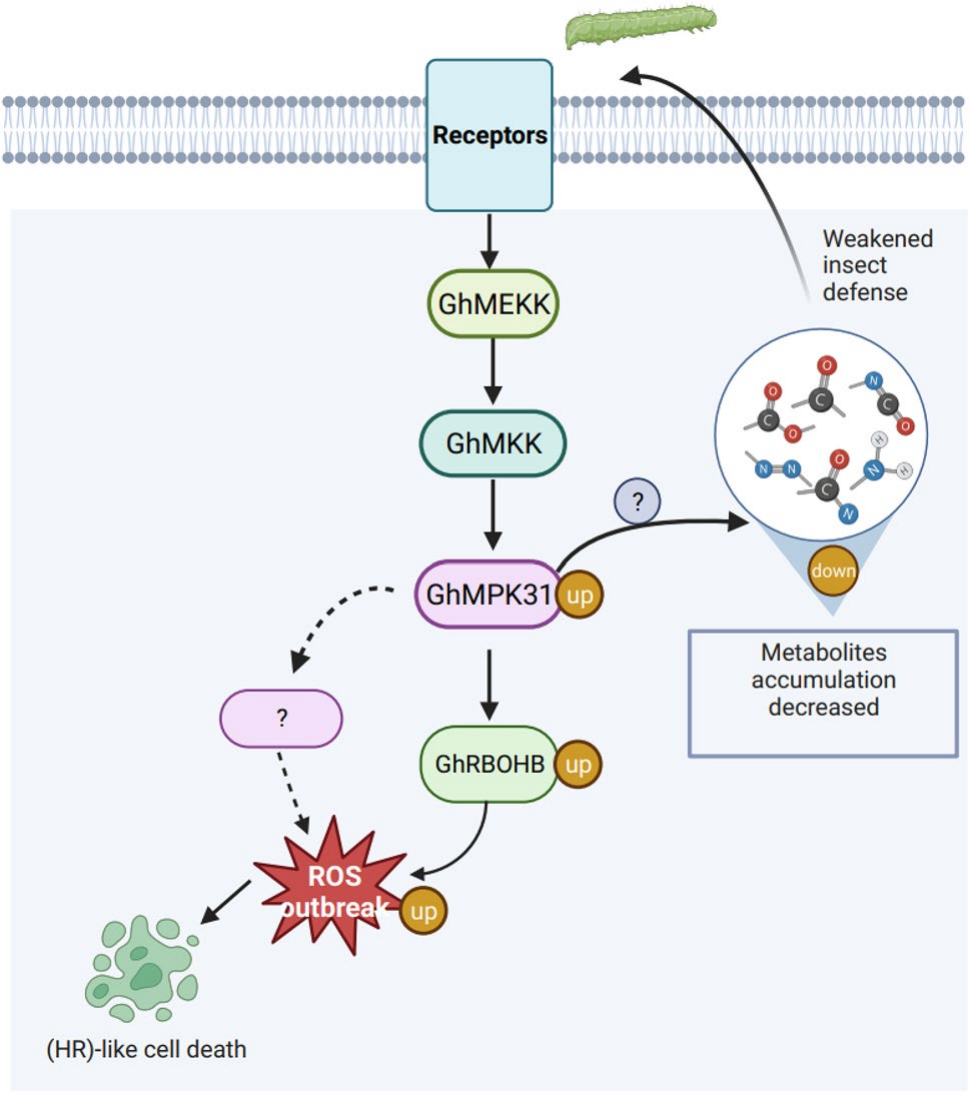
Model of GhMPK31 regulating ROS outbreak and defense decline in upland cotton. Enhanced expression of *GhMPK31* triggers the expression of *GhRBOHB* and other genes, resulting in ROS outbreak and HR-like cell death in plant leaves. Simultaneously, it hampers the accumulation of defense metabolites, leading to a decline in cotton's defense ability against herbivorous insects

In conclusion, this study contributes to the existing knowledge on the mechanism by which *MAPK* regulates the outbreak of ROS, enhancing our understanding of how *MAPK* regulates plant defense against herbivores, we also proposed a model for *GhMPK31* regulation of ROS outbreak and defense decline in cotton plants (Fig. 8). Additionally, it provides a foundation for further investigation into the functional characteristics of the *MAPK* gene family in upland cotton.

### Supplementary Information

Below is the link to the electronic supplementary material.

Supplementary file1 (PPTX 901 KB)

Supplementary file2 (XLSX 1285 KB)

## Data Availability

The RNA-seq data used in this study are available in the NCBI database under the accession PRJNA1087520.
