# Supplementary material for: Comprehensive analysis of MAPK gene family in upland cotton (Gossypium hirsutum) and functional characterization of GhMPK31 in regulating defense response to insect infestation
Source: Plant Cell Rep. 2024 Mar 18;43(4):102. doi: 10.1007/s00299-024-03167-1 (PMC10948490; doi:10.1007/s00299-024-03167-1)
Supplement: Supplementary file 1 — Supplementary file1 (PPTX 901 KB) [file 299_2024_3167_MOESM1_ESM.pptx]

## Slide 1
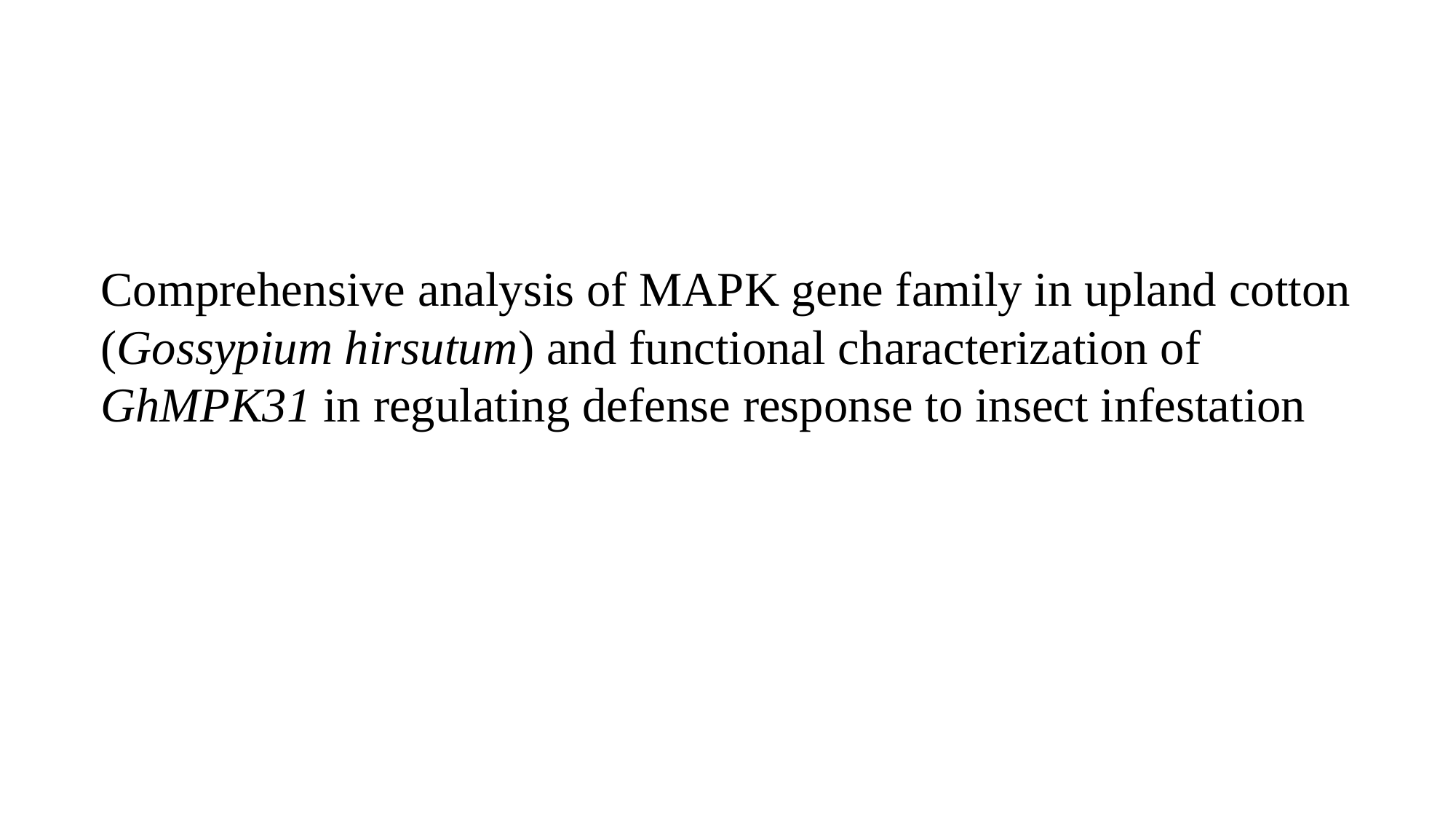

Comprehensive analysis of MAPK gene family in upland cotton (Gossypium hirsutum) and functional characterization of GhMPK31 in regulating defense response to insect infestation

## Slide 2
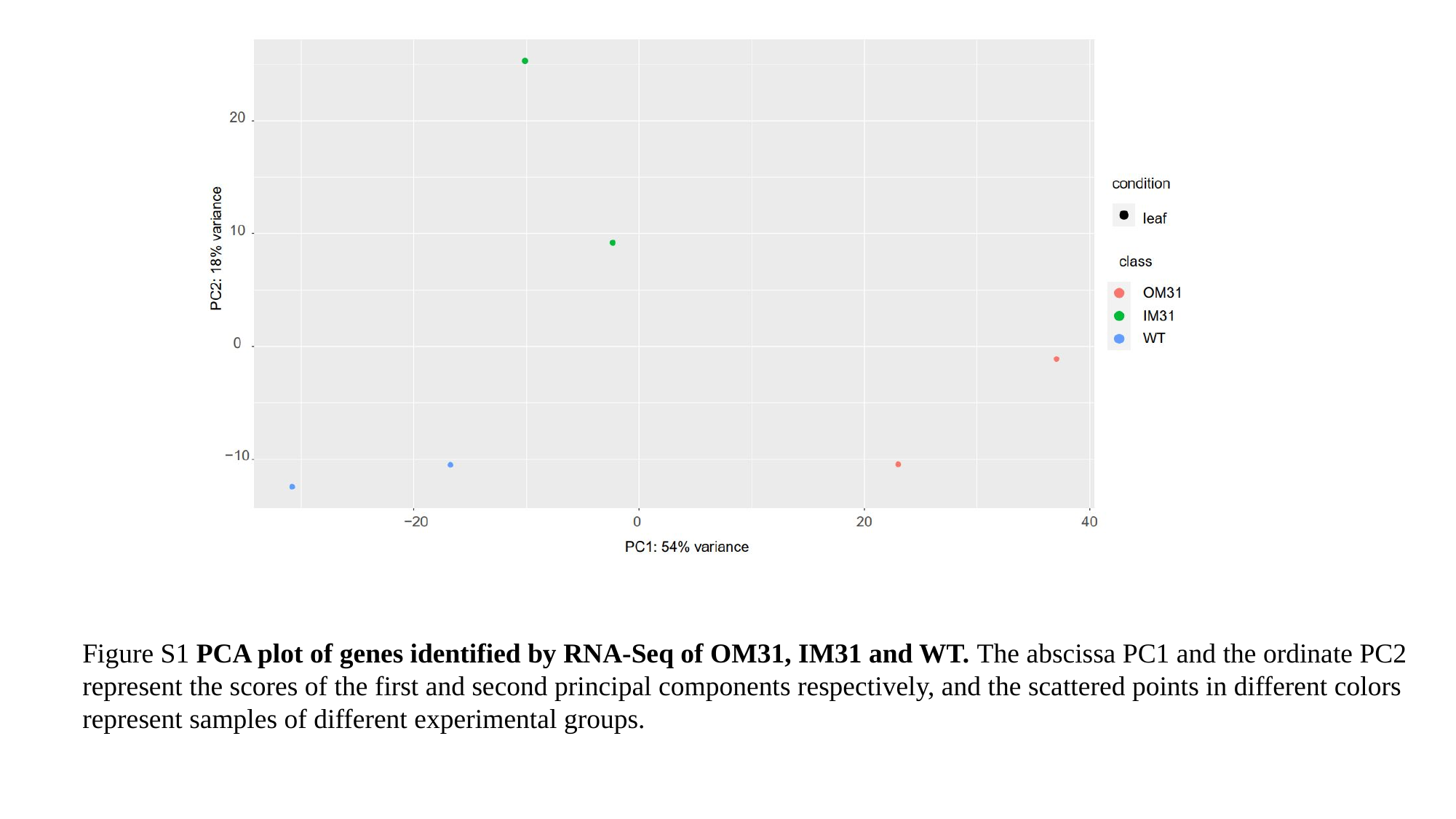

Figure S1 PCA plot of genes identified by RNA-Seq of OM31, IM31 and WT. The abscissa PC1 and the ordinate PC2 represent the scores of the first and second principal components respectively, and the scattered points in different colors represent samples of different experimental groups.

## Slide 3
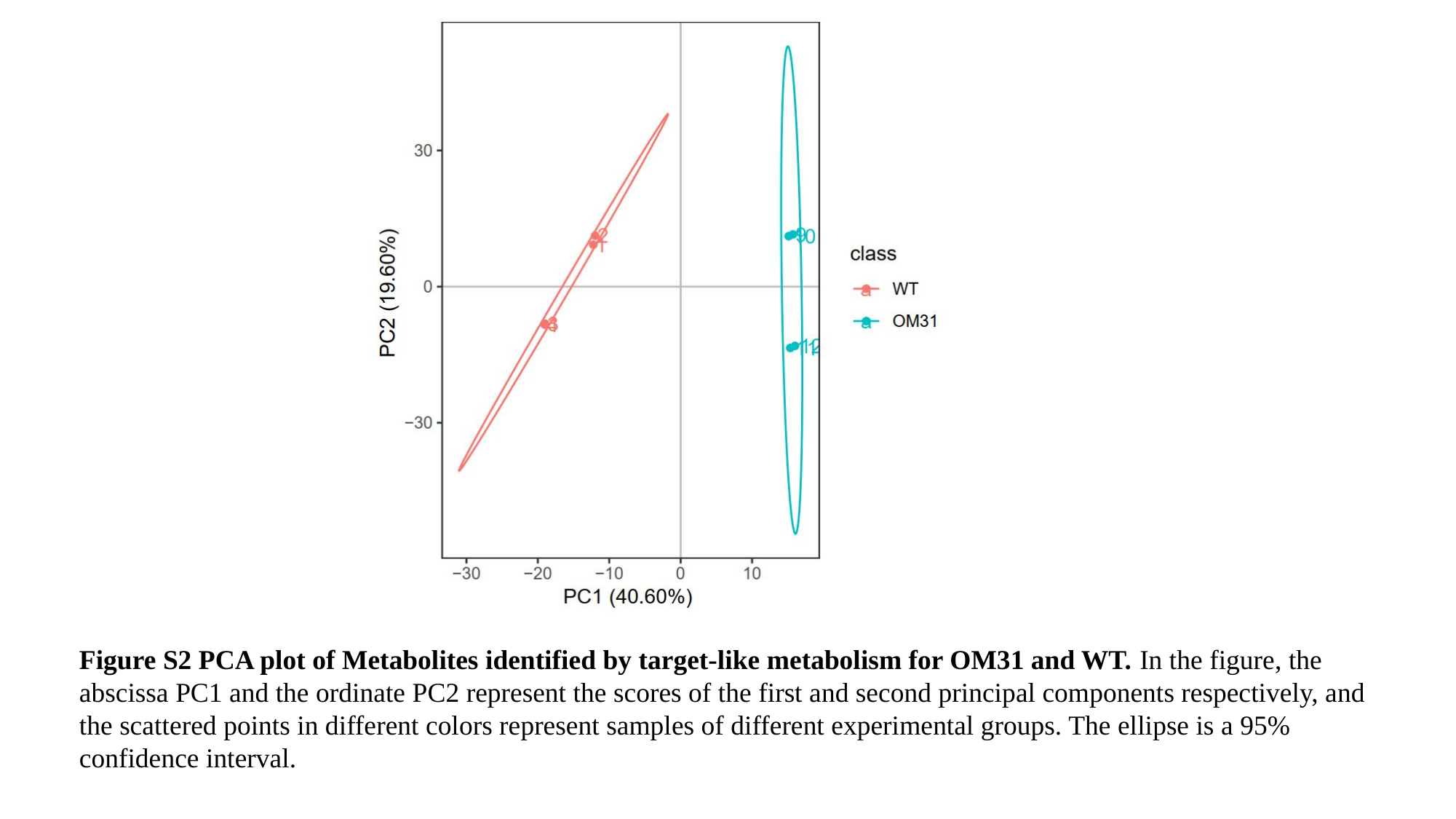

Figure S2 PCA plot of Metabolites identified by target-like metabolism for OM31 and WT. In the figure, the abscissa PC1 and the ordinate PC2 represent the scores of the first and second principal components respectively, and the scattered points in different colors represent samples of different experimental groups. The ellipse is a 95% confidence interval.

## Slide 4
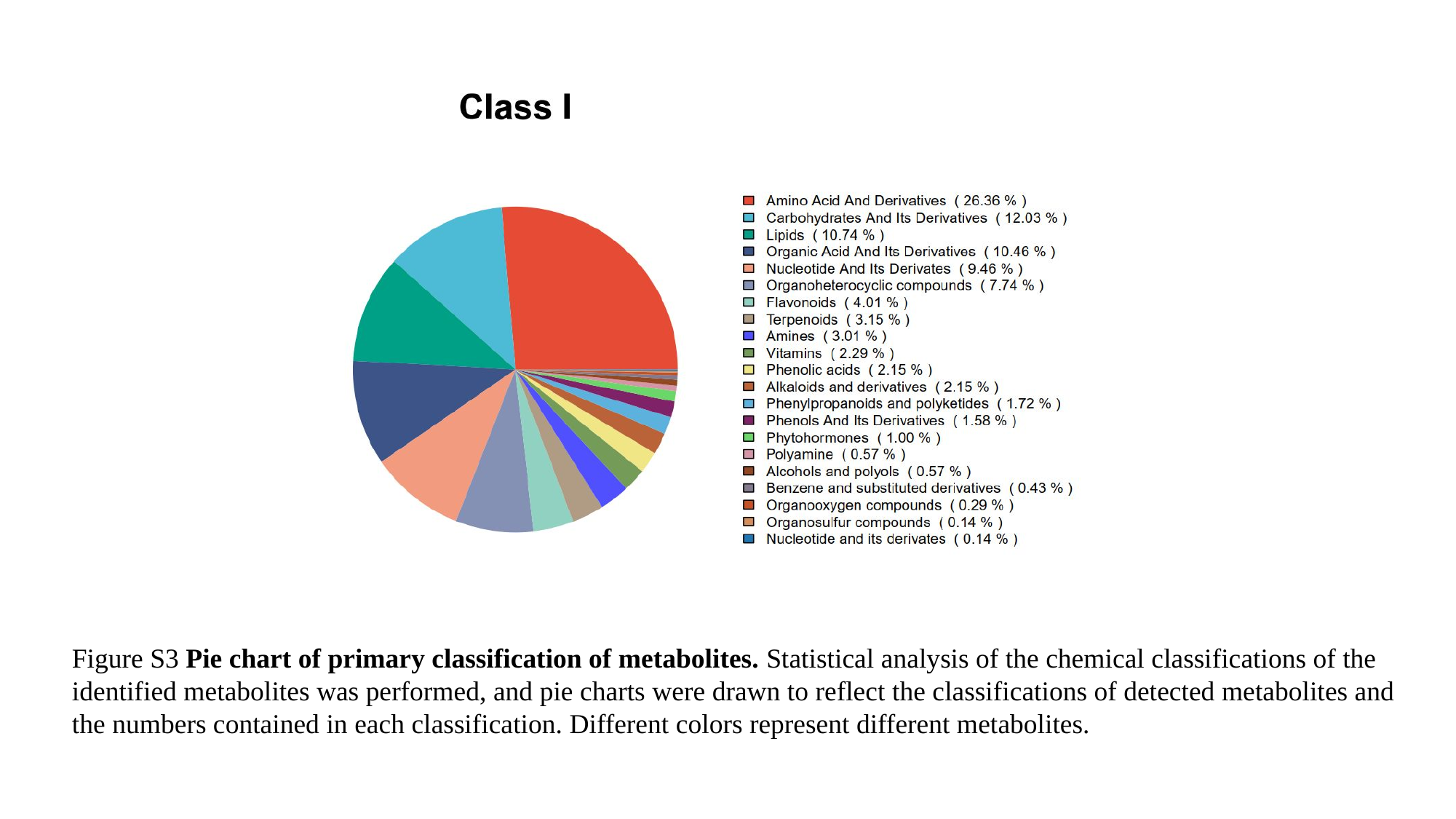

Figure S3 Pie chart of primary classification of metabolites. Statistical analysis of the chemical classifications of the identified metabolites was performed, and pie charts were drawn to reflect the classifications of detected metabolites and the numbers contained in each classification. Different colors represent different metabolites.

## Slide 5
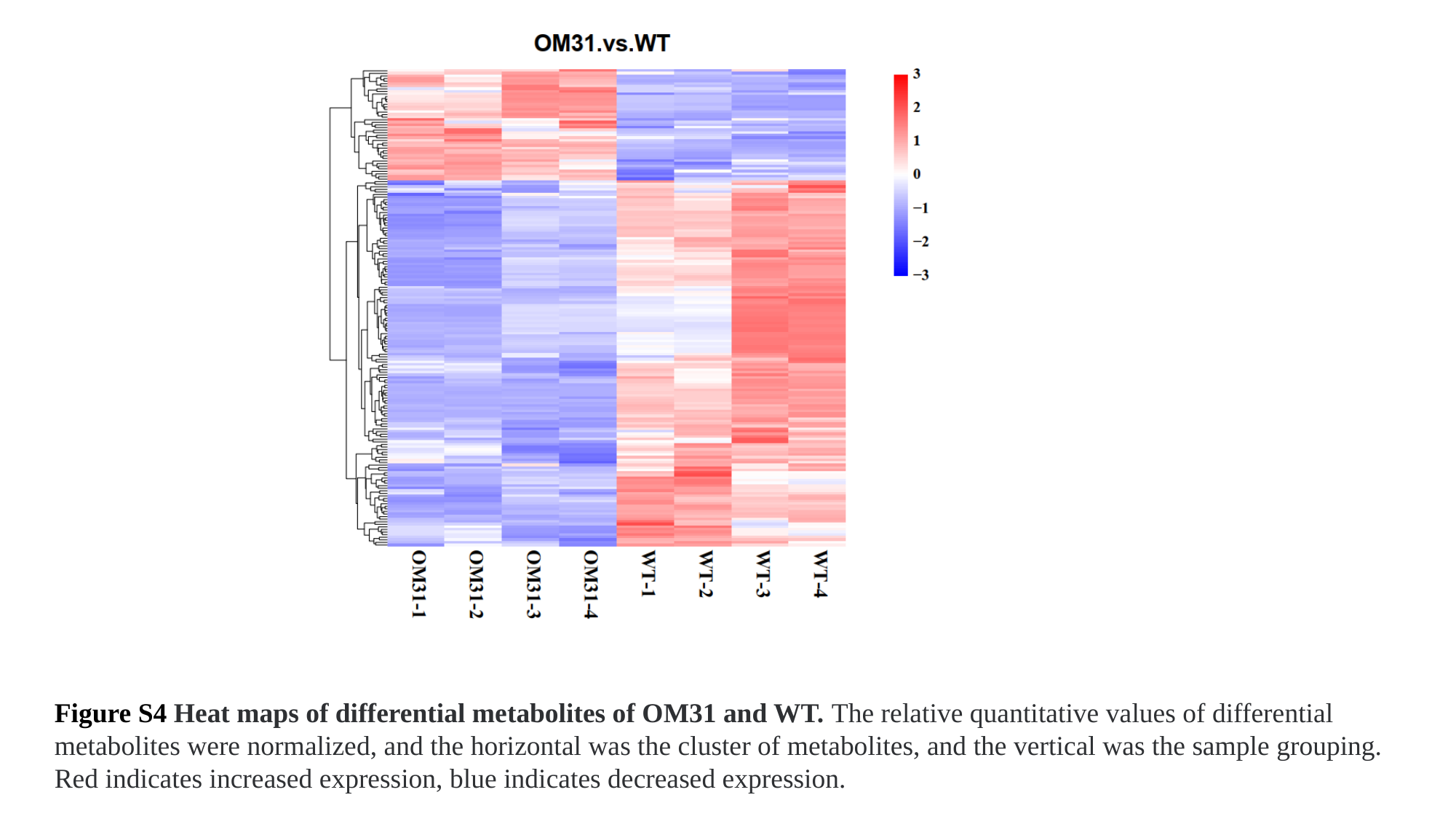

Figure S4 Heat maps of differential metabolites of OM31 and WT. The relative quantitative values of differential metabolites were normalized, and the horizontal was the cluster of metabolites, and the vertical was the sample grouping. Red indicates increased expression, blue indicates decreased expression.

## Slide 6
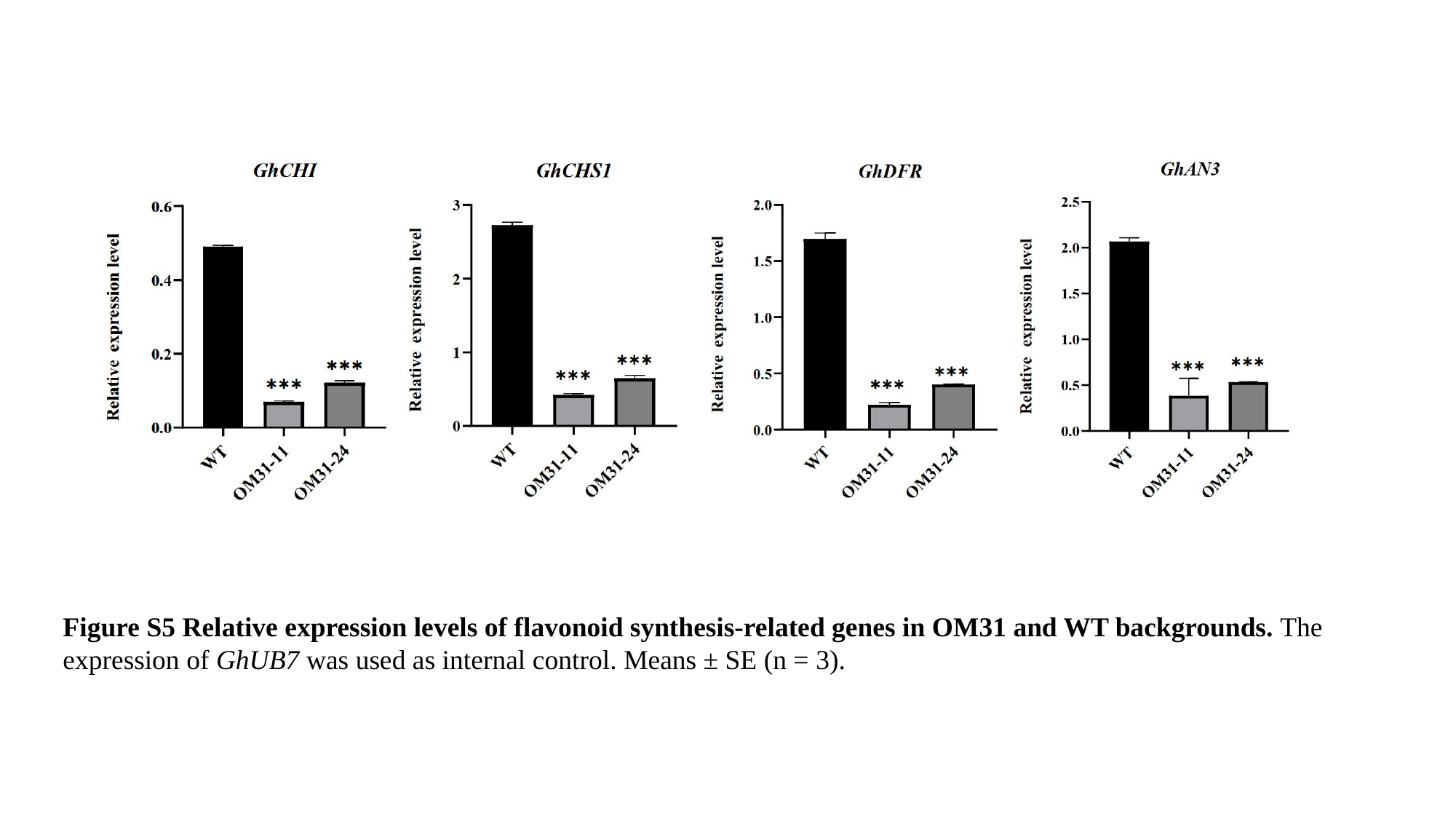

Figure S5 Relative expression levels of flavonoid synthesis-related genes in OM31 and WT backgrounds. The expression of GhUB7 was used as internal control. Means ± SE (n = 3).

## Slide 7
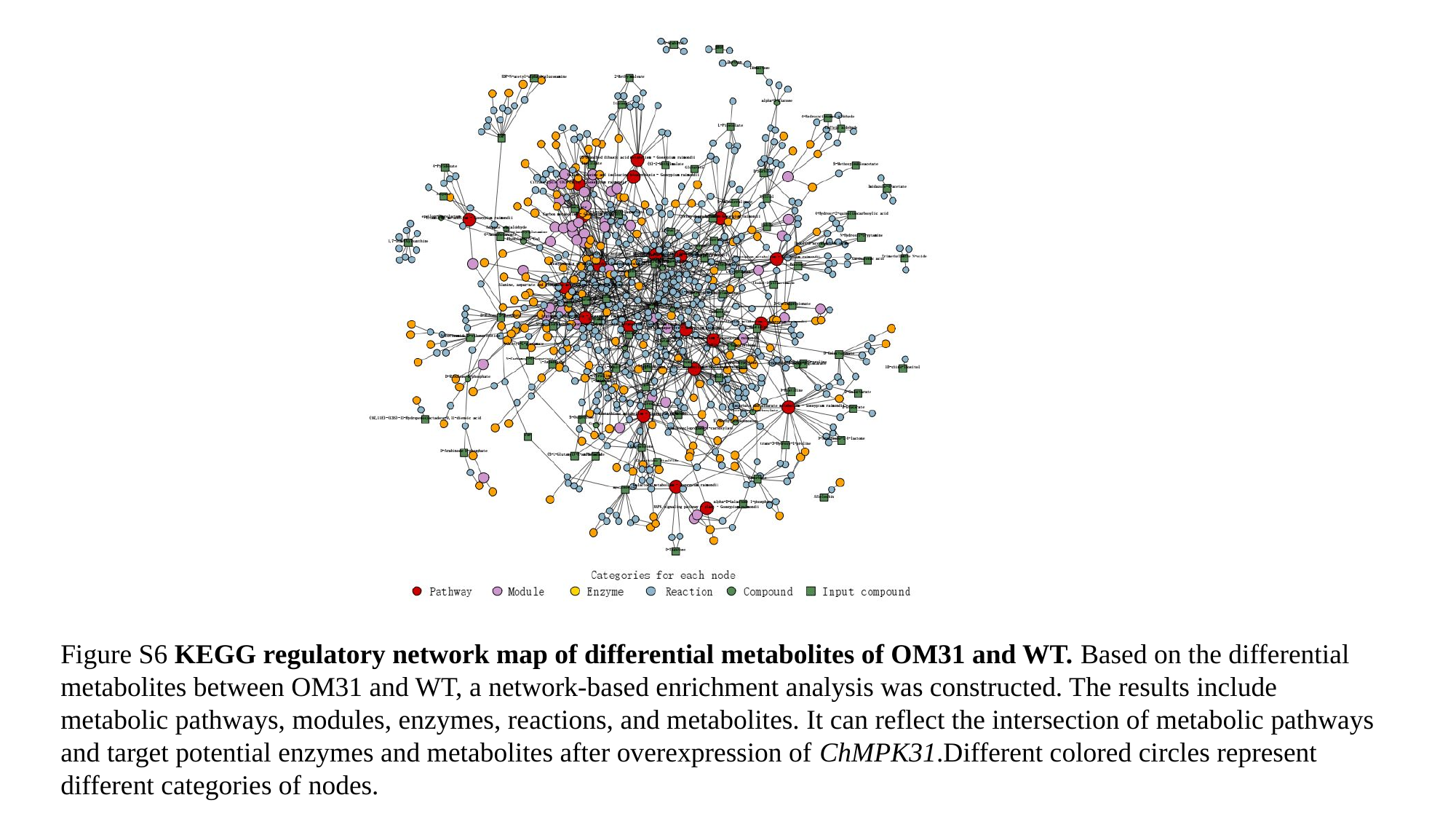

Figure S6 KEGG regulatory network map of differential metabolites of OM31 and WT. Based on the differential metabolites between OM31 and WT, a network-based enrichment analysis was constructed. The results include metabolic pathways, modules, enzymes, reactions, and metabolites. It can reflect the intersection of metabolic pathways and target potential enzymes and metabolites after overexpression of ChMPK31.Different colored circles represent different categories of nodes.
